# Whole mitochondrial genome scan for population structure and selection in the Atlantic herring

**DOI:** 10.1186/1471-2148-12-248

**Published:** 2012-12-22

**Authors:** Amber GF Teacher, Carl André, Juha Merilä, Christopher W Wheat

**Affiliations:** 1 Department of Biosciences, University of Helsinki, P.O. Box 65, FI-00014, Helsinki, Finland; 2 Centre for Ecology and Conservation, University of Exeter, Cornwall Campus, Treliever Road, Penryn, Cornwall, TR10 9EZ, UK; 3 Department of Marine Ecology – Tjärnö, University of Gothenburg, SE-452 96, Strömstad, Sweden; 4 Ecological Genetics Research Unit, Department of Biosciences, University of Helsinki, P.O. Box 65, FI-00014, Helsinki, Finland; 5 Current address: Centre for Ecology and Conservation, Biosciences, College of Life and Environmental Sciences, University of Exeter, Cornwall Campus, Treliever Road, Penryn, Cornwall, TR10 9EZ, UK

**Keywords:** Baltic sea, *Clupea harengus*, Fisheries, Genome, Mitochondria, Selection, Phylogeography

## Abstract

**Background:**

Marine fish, such as the Atlantic herring (*Clupea harengus*), often show a low degree of differentiation over large geographical regions. Despite strong environmental gradients (salinity and temperature) in the Baltic Sea, population genetic studies have shown little genetic differentiation among herring in this area, but some evidence for environmentally-induced selection has been uncovered. The mitochondrial genome is a likely target for selection in this system due to its functional role in metabolism.

**Results:**

We sequenced whole mitochondrial genomes for herring from throughout the Baltic region (n=98) in order to investigate evidence for geographical structuring, selection, and associations between genetic and environmental variation. Three well-supported clades that predate the formation of the Baltic Sea were identified, but geographic structuring of this variation was weak (Φ_ST_ = 0.036). There was evidence for significant positive selection, particularly in the ND2, ND4 and ND5 genes, and amino acids under significant selection in these genes explained some of the clade formation. Despite uncovering evidence for selection, correlations between genetic diversity or differentiation with environmental factors (temperature, salinity, latitude) were weak.

**Conclusions:**

The results indicate that most of the current mtDNA diversity in herring predates the formation of the Baltic Sea, and that little structuring has evolved since. Thus, fisheries management units in this region cannot be determined on the basis of mtDNA variability. Preliminary evidence for selection underlying clade formation indicates that the NADH complex may be useful for examining adaptation and population structuring at a broader geographical scale.

## Background

Marine fish often show weak genetic divergence over large geographical areas [1,2]. This may be due to high larval dispersal and adult migration in marine systems (i.e. high gene flow), and/or because of low genetic drift due to the large effective population sizes of many marine fish [3-5]. The predominant tools used for population genetic studies on marine species have been a small number of microsatellite markers (typically <10 [6]). However, microsatellites derived from genomic libraries are unlikely to be indicative of adaptive differentiation unless they are in linkage with functional genes or located within their coding regions [7]. Thus, in the context of fisheries management, the general lack of focus on molecular markers that are associated with genomic regions under selection suggests that marine populations may be incorrectly managed as panmictic. This is especially true for marine species inhabiting regions where local adaptation to steep environmental gradients is likely to exist [8,9].

Mitochondrial DNA (mtDNA) is a potentially useful source of informative markers for investigating population structuring and adaptation in marine species due to its low effective population size (1/4^th^ of nuclear genes; but see [10]) and documented functional roles in thermal adaptation and aerobic capacity [11,12]. Mitochondria produce up to 95% of eukaryotic cell energy through oxidative phosphorylation, thereby having an important role in metabolic performance. Despite the fact that there is very strong linkage between mitochondrial genes due to which they can not be considered as evolutionarily independent, the genes do differ in their mutation rates [13] and functions. However, selective forces acting on one site will affect the whole mitochondrial genome, such that variation even in non-coding regions will deviate from neutral expectations. As well as selection acting directly on the mitochondrial genome, selection on the nuclear genome may also indirectly influence the mitochondrial genome, as mitochondria-encoded subunits of the respiratory chain are embedded within nuclear-encoded subunits [14]. Furthermore, any maternally inherited factor could in theory influence mitochondrial haplotype diversity [14]. Recombination is thought to be rare (though has been shown to occur [12]), thus increasing the potential for selective sweeps [15].

Several studies have detected specific mutations and regions of the mitochondrial genome that appear to be under positive selection. Mutations in mitochondrial genes have been linked to a large number of human diseases ( http://www.mitomap.org), to thermal adaptation [11,16] and to aerobic capacity [17]. Furthermore, a study of 12 protein-coding mitochondrial genes in 41 placental mammals found evidence for adaptive evolution of the mitochondrial genome in species with specialised metabolic requirements (e.g. large body size such as elephants) and high oxygen requirements (e.g. diving cetaceans, alpacas at high altitudes; [18]); particularly strong positive selection was detected in ND2, ND4, ND5, and ATP8. At the population level, there is some evidence for mtDNA selection in birds (*Parus* spp.), based on the fact that certain populations consistently had a single non-synonymous substitution in the ND2 gene [19]. In addition, walleye Pollock (*Theragra chalcogramma*) have been shown to have more mitochondrial diversity than expected under a model of mutation and genetic drift, indicating that selection is occurring along a North–south gradient in two separate sampling regimes on either side of the North Pacific [20].

Mitochondrial DNA may be a particularly useful marker for marine species, as they are likely to experience strong selective pressures towards high aerobic capacity, and those with wide distribution ranges may also need to adapt to environmental clines [21]. Broader sampling of the mtDNA genome holds the possibility of greater resolution of both the population structure and intraspecific selection dynamics of marine fish. A very small number of studies have directly examined mtDNA selection in marine species. Cytochrome c oxidase (COX 2) has been shown to be under selection in fish with high aerobic requirements (billifishes, but not tunas; [22]). In addition, Garvin *et al. *[23] found evidence for codons under positive selection in the ND2 and ND5 genes in Pacific salmon species, indicating that Complex I (the ND gene complex) has been particularly important in the evolution of these species. In contrast, a global study of killer whale mitochondrial genomes found very high levels of conservation, with only two positively selected codons, both in the cytochrome b gene, and both in Antarctic pack-ice individuals, which may actually represent distinct subspecies [24]. Although there are very few studies of mtDNA selection in marine species, it has been more common to use mtDNA as a marker for population and phylogeographic studies for marine fish. However, the majority of such studies have almost exclusively used only one or two mitochondrial genes or gene fragments from across a very broad geographical scale [25,26].

The Atlantic herring (*Clupea harengus*) in the Baltic Sea provide an ideal case study to investigate mitochondrial genome variation and the possibility of selection, as this geographical region is characterised by strong environmental gradients and local adaptation to this variation may be expected. The Baltic Sea region, including the Skagerrak and Kattegat seas that connect the Baltic Sea to the North Sea, is a post-glacial environment, which has existed only for approximately the past 8,000 years [27]. A salinity gradient occurs from North to South, and East to West, ranging from 1 to 20 PSU (parts per thousand) within the Baltic Sea, and up to 35 PSU at the mouth of the Baltic Sea. Within the Baltic Sea, a temperature gradient also runs from the North to the South ( http://www.itameriportaali.fi/en/itamerinyt/en_GB/itamerinyt/). The Skagerrak and Kattegat seas mark the region where the environment changes from being strictly marine (North Sea) to brackish (Baltic Sea).

Herring in the Baltic region appear to be weakly differentiated, most likely due to high gene flow caused by larval drift and migrating adults, large population sizes, and perhaps also due to young age of the Baltic Sea meaning that there may have been insufficient time for population differentiation to occur. There is support for the Baltic representing a distinct population, with a boundary to gene flow occurring around the Skagerrak/Kattegat Sea region [28-32]. There is also some evidence for weak population structuring within the Baltic Sea, with possible barriers to gene flow separating Rügen (Germany) herring from all other studied Baltic Sea samples, and separating populations in the southern Baltic Sea from those in the North [33]. SNP data shows evidence for introgression of North Sea alleles to the southern Baltic Sea [34]. Microsatellite and SNP allelic frequencies have been shown to correlate with variation in sea surface temperature and salinity within the Baltic Sea, providing preliminary evidence that environmentally-induced selection may be occurring at this geographical scale [32,34-36]. Indeed, there is evidence from tagging experiments on Atlantic herring in the Baltic for a high degree of homing to spawning sites, and very low levels of migration between sites at spawning time [37], enhancing the potential for local adaptation. Furthermore, exome sequencing has demonstrated [36] that many loci identified as likely to be under positive selection are clustered within particular chromosomal regions, and can be assigned to specific candidate genes that are thought to be under salinity-induced selection (e.g. ATPase proton pump which is involved in osmoregulation), providing evidence for adaptation in this system.

Here, we used the whole mitochondrial genome to investigate population differentiation, selection and molecular evolution in the Atlantic herring in the Baltic Sea. We addressed three main questions by analysing a large number (n = 98) of whole mitochondrial genome sequences of herring from 16 localities covering the entire Baltic Sea, and an additional sample from the Barents Sea. First, is there any evidence for geographical structuring of genomic variation? Second, is there any evidence for positive selection acting on the mitochondrial genome? Third, is there any evidence that the genomic variation is associated with local variation in sea temperatures and salinity? We conclude by discussing the implications of our findings for the management of herring fisheries in the Baltic Sea.

## Methods

### Sampling

**Figure 1:**
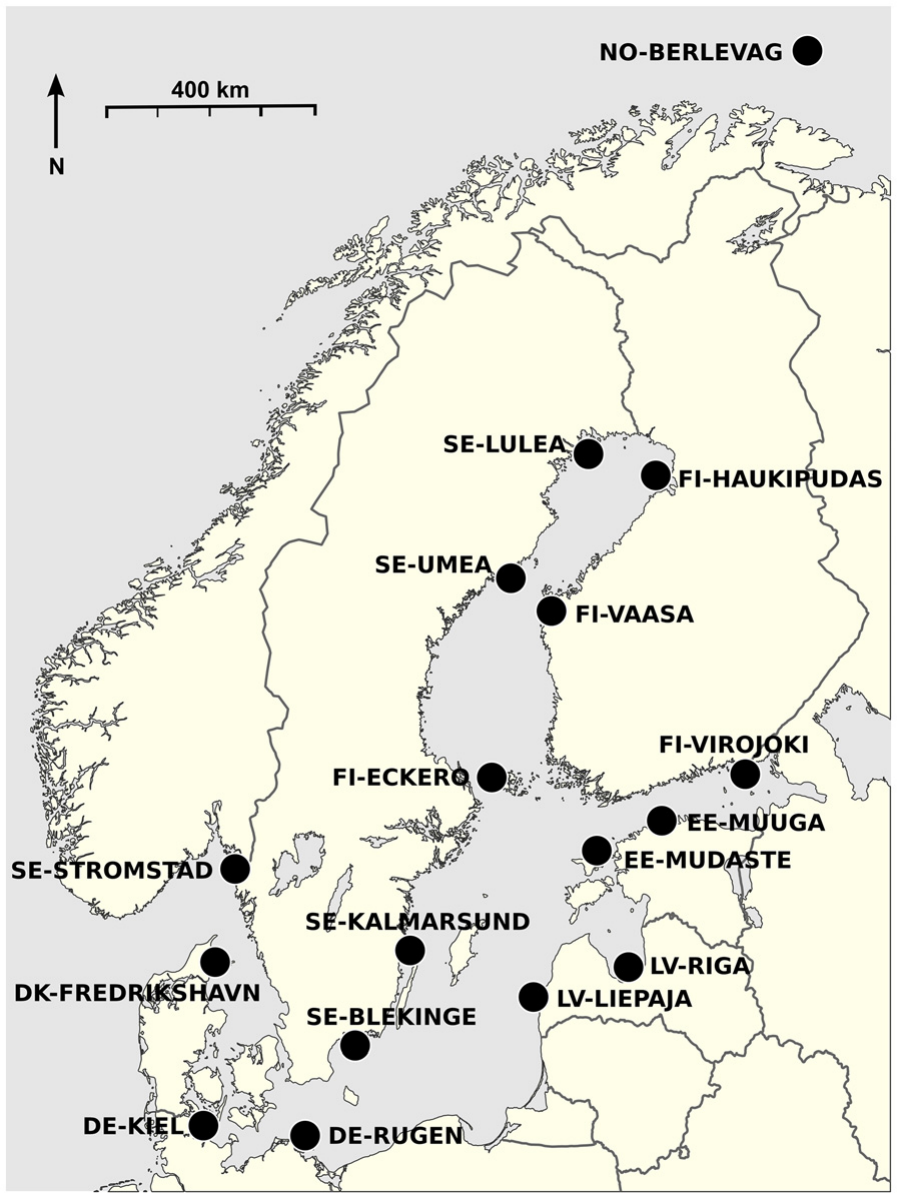
**Map showing sampling locations and site names.** The direction of North and an approximate scale are also shown.

Sampling was designed to cover the whole Baltic Sea, with an additional sample from the Barents Sea (Figure 1, Additional file 1). Herring were fished during the spring spawning seasons of 2009 and 2010, with the exception of the Barents Sea site which were collected in February 2008. In total, we used 98 individuals from 17 sites (5–6 individuals/site; Additional file 1). The samples used in this study were collected in accordance with the national legislation of the countries concerned.

### Sequencing

The mitochondrial genome was amplified in 22 overlapping segments, using 22 novel primer pairs (Additional file 2) which were designed with Primer3 [38] using published *Clupea harengus* sequences as a template (GenBank accession numbers of template sequences: Genome: AP009133; CO1: AM911176, GU324181; Cytb: AF472580, EU224004, EU427557, EU492088, EU492335, EU552605; 16S: X99191, AM911204, DQ912078, DQ983932, EU348305, EU552687, GU324147). PCR mixes for all primer pairs consisted of 5μl Qiagen *Taq* PCR Master Mix (Qiagen, Finland), 3μl H2O, 0.5μl of each primer at 10pmol, and 1μl DNA. The PCR program used for all primer pairs was: denaturation at 95°C for 15 min, followed by 30 cycles of 95°C for 30s, 60°C for 1 min 30s, 72°C for 1 min, followed by a final elongation step at 60°C for 10 min. The resulting products were run on 1% agarose gels to check for amplification, cleaned using Exonuclease I and FastAP (Thermo Fisher Scientific, Finland), and Sanger sequencing was outsourced to Beckman Coulter Genomics (UK) and the Institute for Molecular Medicine (Finland). Sequences were checked manually, assembled, aligned, and annotated in Geneious Pro 5.4 [39]. Annotations were performed using those from a published *C. harengu*s mitochondrial genome (GenBank accession number AP009133). Note that the mtDNA genome has several overlapping genes (the following four pairs of genes overlap: ATP8-ATP6, ATP6-COX3, ND4L-ND4, ND5-ND6), and one gene (ND6) is encoded on the complementary strand. Overlapping nucleotide sections were duplicated so that each gene sequence was fully represented in the individual gene and concatenated gene datasets, and the reverse complement of the ND6 nucleotide sequence was used in order to infer the correct amino acid sequence.

Three dataset types were created (i) whole genome (nucleotide sequence), (ii) each of the 13 individual genes and the control region as separate datasets, (iii) all genes concatenated (i.e. all 13 coding genes concatenated into a single sequence).

### Phylogenetic trees

(i) Whole genome - The most suitable model of nucleotide evolution for the whole genome sequences was chosen using jModelTest 0.1.1 [40,41] This model was coded in to MrBayes [42] to create a Bayesian tree, which was run with two chains for 4.1 million generations, sampling every 100 generations, and discarding 25% of the samples as burnin. The run was stopped once the standard deviation of split frequencies was <0.01, and convergence was checked using Tracer v1.5 [43]. A maximum likelihood consensus tree was calculated for the same dataset using MEGA v.5.05 [44], with 500 bootstraps and using the GTR substitution model. *Clupea pallasii* (GenBank accession number NC009578) was used as an outgroup to root both types of tree. A neighbour-joining tree was produced from the same dataset using between sampling site mean nucleotide distances (proportion of nucleotides at which two sampling sites are different) in MEGA v.5.05 [44]. In order to better visualise the whole genome nucleotide distances, a multidimensional scaling (MDS) plot was generated using the cmdscale function in R [45].

(ii) Individual genes and control region – Datasets for each of the 13 individual coding genes, and for the control region, were run through jModelTest 0.1.1 [40,41]. These models were used as priors in MrBayes [42] to create a Bayesian tree for each gene. MrBayes was run with two chains for two million generations, sampling every 500 generations, and discarding 25% of the samples as burnin. For each gene the standard deviation of split frequencies was <0.02 when the analysis was terminated. *Clupea pallasii* (GenBank accession number NC009578) was used as an outgroup to root all trees.

(iii) All genes concatenated - Trees were also created using the dataset of all coding genes combined. A Bayesian tree was created in MrBayes [42] partitioning the dataset into each of the 13 separate genes, and using individual models for each gene as chosen by jModelTest. MrBayes was run for six million generations, sampling every 500 generations, and discarding 25% of the samples as burnin. *Clupea pallasii* (GenBank accession number NC009578) was used as an outgroup to root the tree. An additional maximum likelihood tree was produced using amino-acid data for all genes concatenated with 500 bootstrap replicates. A neighbour-joining tree was produced with the same dataset, using between sampling site mean amino acid distances (proportion of amino acids at which two sampling sites differ) in MEGA v.5.05 [44]. Also, a multidimensional scaling (MDS) plot was generated using the cmdscale function in R [45] for the all genes concatenated amino acid data.

### Clade dating

Estimating the Time to Most Recent Common Ancestry (TMRCA) of *C. harengu*s, and its intraspecific subclades was performed using a Bayesian relaxed clock method implemented in BEAST v.1.6 [46]. The all genes concatenated dataset was used, with two additional aligned sequences from each of the two nearest species having whole mtDNA genome sequence available ( [47]; *C. pallasii* GenBank accession numbers NC009578, AP009134; *Sprattus sprattus* AP009234, NC009593). Two nodes were used for temporal calibration based upon the phylogenetic relationships among 25 Clupeiforme mtDNA genomes [47]. Nothing is known about the TMRCA between *Sprattus* and *Clupea,* thus a uniform prior between 60 and 10 millions of years ago (Mya) was chosen, with an initial value of 30 Mya. The TMRCA between *C. harengus* and *C. pallasii* was defined as a truncated normal distribution having a mean of 4 Mya, a standard deviation of 1 My, and an upper and lower limit of 3 and 5 Mya, respectively, based on previous estimates for the age of this node [48]. The analysis used the SRD06 model of codon evolution (i.e. HYK with estimated base frequencies, a gamma site heterogeneity model with four categories, and two data partitions; codon sites 1+2, and 3), and an uncorrelated log normal (UCLN) relaxed clock model of evolutionary change. The UCLN mean rate prior was modelled as a uniform distribution from 0 to 60, with an initial value of 9.2 E – 4. The tree prior was set to a Yule speciation process, starting with a randomly generated tree. Multiple analyses were conducted for at least 50 × 10^6 ^iterations each, with a burn-in of 10^6^. Samples were taken every 1000 iterations and operators were optimized automatically. Results of replicate runs, convergence, asympotic performance and effective sample size (ESS) of posterior parameters (> 100 in all cases) were assessed using Tracer v1.5 [43].

### Population structure

Analysis of Molecular Variance (AMOVA) was performed for the whole genome nucleotide dataset, for each individual coding gene, and for the control region, using Arlequin version 3.11 [49] with 10,000 permutations, in order to test the null hypothesis of no population structure. The F_ST _estimator used was Φ_ST_, a direct analogue of Wright's F_ST _for nucleotide sequence divergence. Again, we applied the sequential Bonferroni correction, and a false discovery rate of 0.05. Arlequin was also used to calculate pairwise Φ_ST _for the whole genome and any genes that indicated significant population structure.

Isolation by distance tests to compare genetic and geographic distances were performed using Mantel’s tests (Pearson method) in R [45], using 10,000 permutations. The following datasets were used: Whole genome nucleotides, all coding genes concatenated nucleotide and amino acid data, each individual gene nucleotide and amino acid data, and the control region nucleotide data. The genetic distances were calculated as the number of substitutions per site averaging over all sequence pairs between populations, and calculated using MEGA v.5.05 [44]. Estimates for the geographic distance between each pair of sites using the most direct marine route were calculated using Google Earth [50]. This analysis was performed excluding the Barents Sea reference population (NO-BERLEVAG). We applied two different corrections for multiple testing: the sequential Bonferroni correction [51], and a false discovery rate of 0.05 [52] using the p.adjust function in R [45].

### Selection

Selection is often assessed by looking at the ratio of nonsynonymous (dN) to synonymous mutations (dS), where a ratio of dN:dS > 1 is taken to indicate positive selection [53]. However, this approach is not appropriate for conserved genes in which most mutations are synonymous and a very small number of changes can still be adaptive [54,55]. This approach is also sensitive to sampling strategy as shared ancestry can generate spurious results. Because of these issues, we used TreeSAAP [56] which compares the observed physiochemical amino acid changes inferred from a phylogenetic tree with the expected random distribution of amino acid changes selective neutrality. TreeSAAP compares amino acid sequences in the context of the given tree to infer amino acid replacements, which are then analysed using models that estimate the distributions of 31 physiochemical amino acid properties, under the assumption that under neutrality, every feasible amino acid replacement is equally likely. Although within-site sample sizes are low, it is worth noting that the TreeSAAP analysis is not based on mutation frequencies within populations, but upon the sequences themselves, and so is less affected by sampling strategy than approaches such as dN:dS ratios [56]. We used the dataset for all genes concatenated with the corresponding Bayesian tree, and a sliding window of 20. In order to detect strong positive selection, we only considered most radical amino acid property changes (Categories 6, 7 and 8 on a scale of 1–8 where the lowest categories indicate stabilising selection and the highest categories indicate destabilising selection; [56]) at P ≤ 0.001. The Z scores for the physiochemical properties with significant changes in these categories were plotted. Positive Z scores indicate positive selection, while negative Z scores indicate negative selection. The total number of codons inferred to be under significant positive selection were counted within each gene and each sampling location. The most significant amino acid changes detected were mapped onto the Bayesian tree.

### Environmental correlates of genetic diversity and genetic distance

Mean sea temperature and mean salinity were calculated for 5–10 m of depth from Helcom data ( http://ocean.ices.dk/Helcom/Default.aspx) for locations closest to the sample sites. Two averages were calculated: mean April values, and mean spawning time values. Mean temperatures and salinities for the month of April were used in order to give an estimate of how temperatures varied between sites at a fixed time-point. April was chosen as the representative month as spawning took place from February through to July. In addition, mean temperature and salinity values were obtained for a two-week period spanning the spawning date, with the sampling date taken as the midpoint and one week either side to allow for some degree of error.

Latitude and the distance from the entrance to the Baltic Sea were used as explanatory variables for genetic diversity. The distance from the entrance to the Baltic Sea was calculated as the shortest marine route from each site to a point at the region where the Baltic Sea meets the North Sea (coordinates 057º06’12.85”N, 008º00’59.28”E).

(i) Amino acid and nucleotide diversity

In order to search for correlations between population amino acid or nucleotide diversity (as calculated in MEGA v.5.05 [44] excluding the Barents Sea reference site NO-BERLEVAG) and environmental variables, general linear models were implemented in R [45], with the expectation that genetic diversity may be lower in more extreme environments. Nucleotide diversity and amino acid diversity for all genes concatenated and for each individual coding gene, and nucleotide diversity for the whole genome and the control region, were fitted as response variables in separate models. In each case, the explanatory variables were: distance from the entrance to the Baltic Sea, latitude, mean April temperature, mean April salinity, spawning time temperature, and spawning time salinity. In addition, we included the biologically relevant interaction terms mean April temperature × mean April Salinity, and spawning time temperature × spawning time salinity. Residuals were tested for normality, and each term was dropped individually using the ‘drop1’ function to remove the effect of the order in which the terms were entered in the model. We applied a sequential Bonferroni correction and a false discovery rate of 0.05, and these corrections were applied within each environmental variable, and separately for nucleotides and amino acids.

(ii) Amino acid and nucleotide distance

In order to search for correlations between genetic distance and environmental distance, Mantel’s and partial Mantel’s tests were implemented in R [45]. Genetic distance matrices were calculated for nucleotide and amino acid sequence data for all genes concatenated and for each individual coding gene, and for nucleotide sequences for the whole genome and the control region (total = 30 matrices). The genetic distances were calculated as the number of substitutions per site averaged over all sequence pairs between populations, using MEGA v.5.05 [44]. Distance matrices were generated using R (dist function) for the following variables: mean April temperature, mean April salinity, spawning time temperature, spawning time salinity, and latitude. Mantel’s tests were performed in R (mantel function) between each of the 30 genetic distance matrices and each of the five environmental distance matrices (150 tests). In addition, these 150 tests were repeated as partial Mantel’s tests (mantel.partial function) controlling for geographical distance by sea, in order to remove the effect of isolation by distance from the correlation tests. In all cases, the Pearson correlation coefficients were used, with 10,000 permutations. We applied a sequential Bonferroni correction and a false discovery rate of 0.05; the corrections were applied within each environmental variable, separately for nucleotides and amino acids, and separately for Mantel and partial Mantel tests.

## Results

In total, 98 mtDNA genomes were fully sequenced, each comprised 16,700 base pairs, which corresponded to 3,808 amino acids when all 13 transcribed genes were concatenated. No identical sequences were encountered. Annotated genomes were submitted to GenBank (accession numbers: KC193680 - KC193777). Basic information for each coding gene is included in Additional file 3.

### Phylogenetic trees

**Figure 2:**
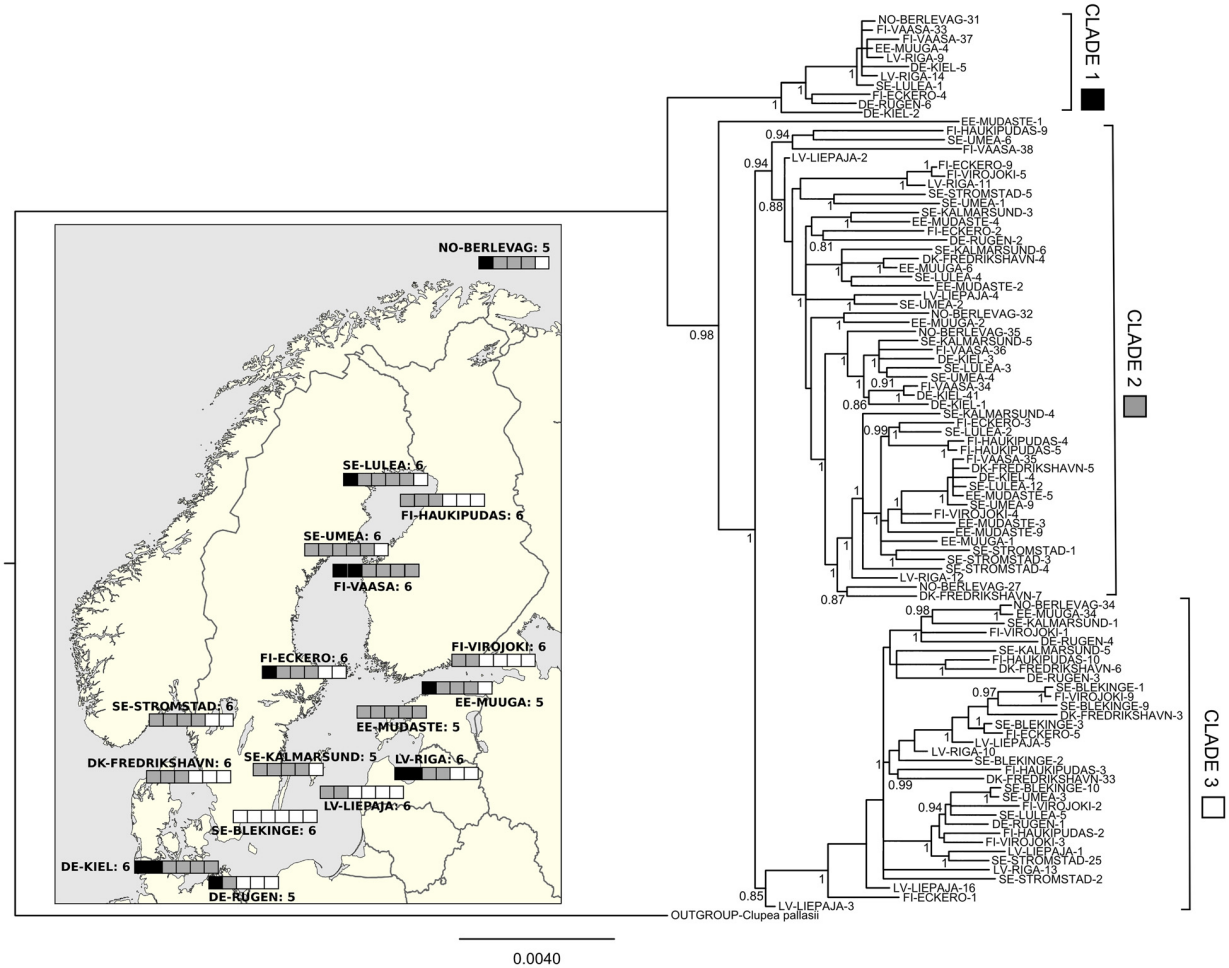
**Bayesian tree for whole genome nucleotide sequences.** Posterior probability values of over 0.8 for node support are shown. Inset is a map showing the Bayesian tree clades. Each block of squares represents one sampling site (accurate points for each sampling site can be seen in Figure 1), and each square represents one individual at that sampling site. Black squares represent clade 1, grey squares clade 2, and white squares clade 3. The name of the sampling site is shown next to the blocks, and the number of samples is shown alongside. Site EE-MUDASTE actually has six samples, but only five are shown as sample EE-MUDASTE-1 is an outlier on the Bayesian tree.

**Figure 3:**
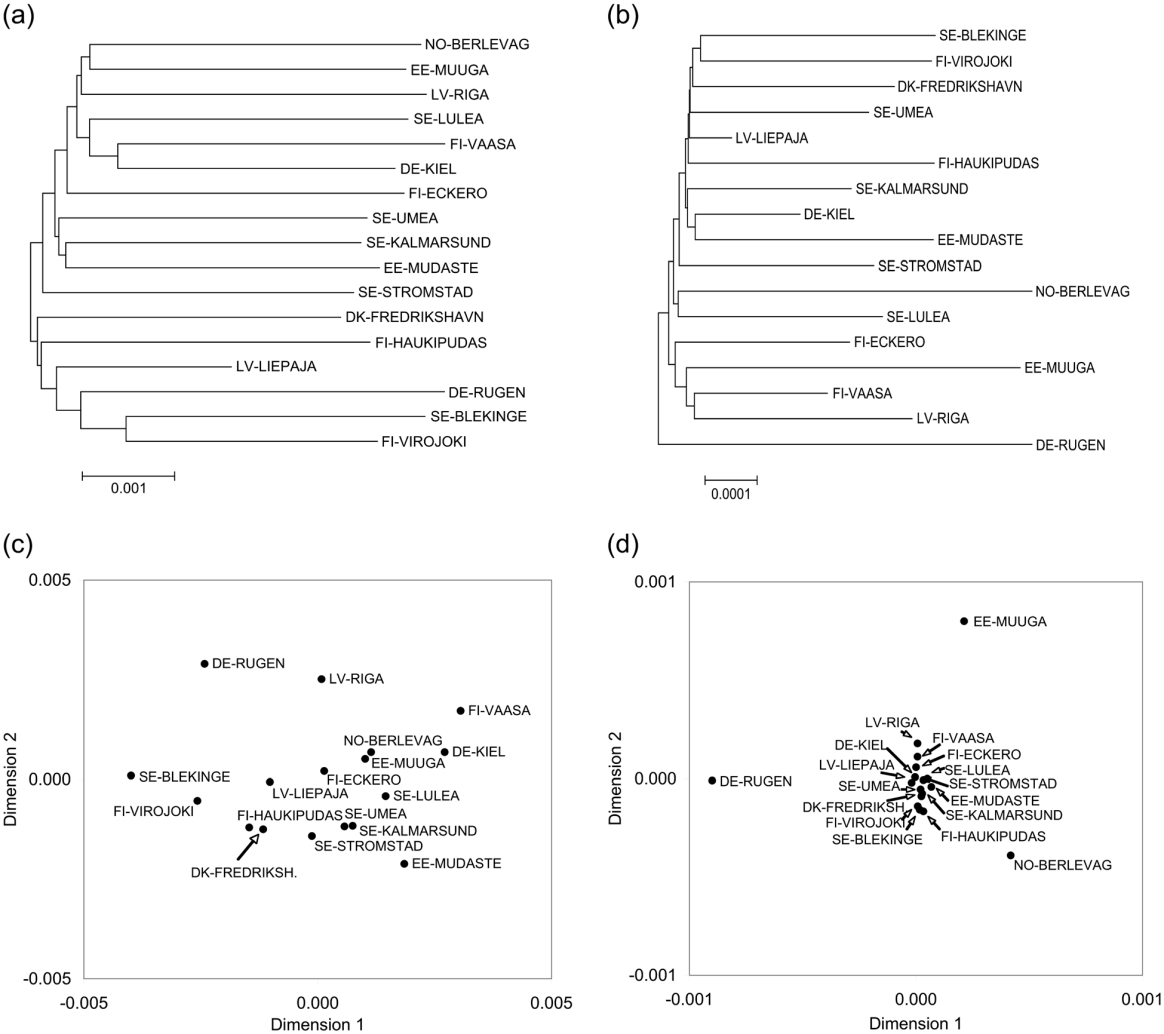
**Un-rooted Neighbour Joining trees and multidimensional scaling plots.** The Neighbour Joining trees were produced using between-group **(a)** whole genome nucleotide distances, and **(b)** all coding genes concatenated amino acid distances. The multidimensional scaling plots were produced using **(c)** whole genome nucleotide distance, and **(d)** all genes concatenated amino acid distance.

(i) Whole genome - The jModelTest results for all analyses can be found in Additional file 3. The Bayesian tree revealed three well-supported main clades, with one outlier (sample EE-MUDASTE-1; Figure 2). The maximum likelihood tree showed the same main three clades as the Bayesian tree, with high bootstrap support (data not shown). The three main clades (based on the Bayesian tree) did not cluster geographically (Figure 2). The neighbour-joining tree using between site nucleotide distances did not show any obvious clades or geographic structuring (Figure 3). Likewise, the multidimensional scaling plot revealed no population structuring (Figure 3).

(ii) Each gene individually and control region – Some Bayesian trees for individual coding genes (ATP6, ATP8, ND3 and ND4L) showed no resolution. However all other genes and the control region showed clustering that was consistent with the three main clades identified in the whole genome tree (data for individual genes not shown).

(iii) All genes concatenated - The partitioned Bayesian nucleotide tree showed identical major clades to the whole genome Bayesian tree. The maximum likelihood tree based on amino acids for all coding genes showed no well supported clades (the highest bootstrap support value was 65%, data not shown), though the samples tended to cluster similarly as in the nucleotide-based trees. The neighbour-joining tree using between site amino acid distances showed no obvious clades, and no clear geographic structuring. However, DE-RUGEN appears to stand out at the root of the tree (Figure 3). The multidimensional scaling plot based on the all genes concatenated amino acid data shows that DE-RUGEN, NO-BERLEVAG, and EE-MUUGA are quite different from all the other sites, and also from each other (Figure 3).

### Clade dating

The mean TMRCA for the *C. harengus* sequences was 2.82 Mya (95% Highest Posterior Density (HPD): 1.95 - 3.67), and between subclades 2 and 3 the mean TMRCA was 1.97 Mya (95% HPD: 1.37 – 2.64). The posterior estimate of the mean rate of evolutionary change, for the concatenated gene set over the whole tree, was 0.0047 substitutions/site/Myr (95% HPD: 0.0035 – 0.0060), with little rate variation across temporal scales within the *Clupea* species lineages (results not shown). Given the large uncertainty in the realized rate variation across temporal scales, which a growing body of studies suggests increases from 2 to 6 X in comparisons between within population and interspecific levels [57-59], we also assessed our results assuming a 10X higher rate of evolutionary change. Using a 10X higher rate provides a robust lower limit on the TMRCA and decreases the aforementioned TMRCA estimates and their 95% HPDs by a factor of 10 (e.g. the mean TMRCA of the *C. harengus* clade is 282 Kya, and for the two subclades 197 Kya). Analysis of the posterior distributions for the constrained nodes indicates that the TMRCA age estimates are highly influenced by the prior constraints, indicating the need for more detailed study of age estimates for these clades and the Clupeiformes as a whole before robust temporal insights can be obtained.

### Population structure

The AMOVA using the whole genome dataset gave an overall Φ_ST_ = 0.036 (P = 0.022 ± 0.004). Looking at individual coding genes and the control region, overall Φ_ST_ across populations ranged from 0 to 0.105, with ATP8 showing the lowest and COX2 the highest value (Additional file 4 and Additional file 5). On application of sequential Bonferroni correction, or a false discovery rate of 0.05, only COX2 was significant.

Pairwise Φ_ST _estimates indicated significant population structuring between many population pairs in the whole genome nucleotide analysis (Additional file 6), with population SE-BLEKINGE being the most distinct in this dataset (this is supported by the MDS plot, Figure 3). In addition, the COX2 pairwise Φ_ST _analysis also indicated SE-BLEKINGE to be the most differentiated population, but showed fewer other significant pairwise results than the whole genome nucleotide analysis.

Isolation by distance tests showed a significant correlation between ATP8 nucleotide distances and distance by sea (r = 0.282, n = 16, P = 0.030), and borderline significant correlations between ATP8 amino acid distances (r = 0.271, n = 16, P = 0.060) and ATP6 nucleotide distances (r = 0.210, n = 16, P = 0.064) with distance by sea. No further statistically significant isolation by distance results were detected (Additional file 7). All tests were non-significant after correcting for multiple tests.

### Selection tests

**Figure 4:**
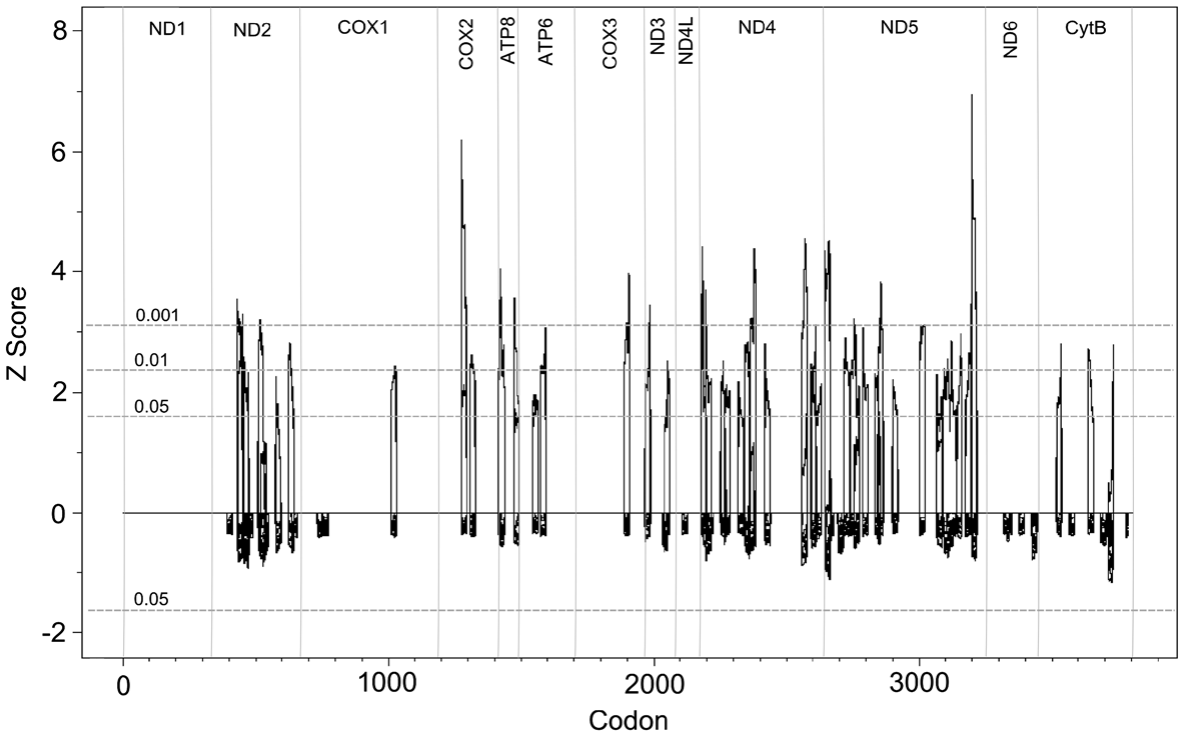
**TreeSAAP results showing regions of the mitochondrial genome under positive disruptive selection.** The confidence intervals for P = 0.05, 0.01, and 0.001 are shown with horizontal dashed grey lines, and each coding gene is marked off with vertical solid grey lines.

**Figure 5:**
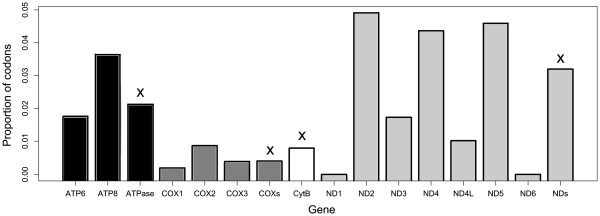
**Bar chart showing the proportion of codons under significant positive disruptive selection in each gene and complex.** The four complexes are marked with an ‘x’ above the column.

TreeSAAP detected significant (P ≤ 0.001) changes in amino acid physiochemical properties in many parts of the herring mitochondrial genome (Figure 4). The highest proportions of codons under selection were found in the ND2, ND5, ND4 and ATP8 genes respectively (Figure 5), and in the sampling sites DE-RUGEN, EE-MUUGA, and NO-BERLEVAG (Additional file 8). Two codons showed particularly significant results (Figure 4, COX2 and ND5 peaks), however when these amino acid changes were mapped to the Bayesian tree, they were found to occur only on terminal branches and did not cluster together. The COX2 change only occurred in a single individual, and the ND5 change only occurred in four individuals (two from clade 2, and two from clade 3). Due to the small within-site sample sizes and associated sampling variance, the importance of these terminal mutations can not be verified. Of the significant amino acid changes that occurred in internal branches, two were found in all but one sample in clade 1 (one in each of the ND4 and ND5 genes), and another was found in all samples in clades 2 and 3 (in the ND2 gene). Unlike the amino acid changes occurring in the terminal branches, amino acid changes in the basal areas of the tree will not be influenced by sampling variance.

### Environmental correlates of genetic diversity and distance

As our pairwise Φ_ST _estimates indicated significant population structuring between many population pairs, we proceeded with correlation tests between genetic and environmental diversity.

(i) Amino acid and nucleotide diversity

The general linear models indicated little or no correlation between genetic diversity and any of the environmental variables used (distance from entrance to the Baltic, latitude, spawning temperature and salinity, April temperature and salinity; see Additional file 9). Four significant results were obtained; ATP6 amino acid diversity with latitude (F_1,8_ = 6.349, P = 0.040), ATP8 nucleotide diversity with spawning temperature (F_1,8_ = 6.913, P = 0.034) and with the interaction term spawning temperature x salinity (F_1,8_ = 5.866, P = 0.031), and COX3 amino acid diversity with spawning temperature (F_1,8_ = 5.805, P = 0.047). However, on inspection of the data, the ATP6 and COX3 amino acid diversity for the majority of the sites (14 and 15 sites respectively, out of the 16 sites used for this analysis) is zero. No correlations were significant after the application of sequential Bonferroni correction or at a false discovery rate of 0.05.

(ii) Amino acid and nucleotide distance

The Mantel tests correlating genetic distance with environmental distance showed significant associations between the following: ATP6 nucleotide distance and spawning salinity (r = 0.312, P = 0.047), ATP8 nucleotide distance and spawning salinity (r = 0.381, P = 0.043), ATP8 amino acid distance and April salinity (r = 0.404, P = 0.035), COX1 amino acid distance and spawning salinity (r = 0.387, P =0.039), COX1 amino acid distance and April salinity (r = 0.385, P = 0.039), ND3 nucleotide distance and spawning temperature (r = 0.390, P = 0.021) as well as latitude (r = 0.368, P = 0.007), and ND4L nucleotide distance and spawning temperature (r = 0.313, P = 0.030; Additional file 10). When these tests were repeated as partial mantel tests, controlling for geographical distance, the following remained significant: COX1 amino acid distance and spawning salinity (r = 0.408, P = 0.041), COX1 amino acid distance and April salinity (r = 0.414, P = 0.033), ND3 nucleotide distance and spawning temperature (r = 0.388, P = 0.022) as well as latitude (r = 0.373, P = 0.013), and ND4L nucleotide distance and spawning temperature (r = 0.314, P = 0.029). The correlations between ATP6 and ATP8 with salinity were simply due to isolation by distance. No correlations were significant after the application of sequential Bonferroni correction or at a false discovery rate of 0.05.

## Discussion

Our results show that there are three well-supported clades of Atlantic herring in the Baltic Sea region, dating to a time long before the formation of the Baltic Sea. The three clades are all found throughout the Baltic Sea, and also at a site in the Barents Sea, with no obvious geographical clustering. Isolation by distance tests uncovered only borderline significant results for the ATP6 and ATP8 genes, before correcting for multiple testing. Despite this lack of geographical clustering, there was evidence for significant population structuring (whole genome Φ_ST_ = 0.036), and when considering amino acids, three sampling sites were divergent (DE-RUGEN, NO-BERLEVAG, EE-MUUGA). Significant evidence for positive selection was found, particularly in the ND2, ND4, ND5 and ATP8 genes. Amino acid changes associated with selection were found at the base of the major clades clades, and occur more often in the three divergent sampling sites. Despite the evidence for selection, there was little evidence for correlations between genetic diversity or differentiation with environmental factors.

### Phylogeography and clade dating

The three main clades were well supported, and a similar pattern of clustering was found within all genes with adequate resolution. Using interspecific calibrations, the last divergence among these clades (i.e. the split between clades 2 and 3) occurred around 1.37 Mya. In order to provide a robust lower limit to this last divergence event, we also used a 10X faster rate of molecular evolution, which puts the lower limit to 137 Kya years ago. While there is a large degree of uncertainty regarding our temporal estimations of these clades, given that the Baltic Sea is a post-glacial environment that is roughly 8,000 years old [27], the three main *C. harengus* clades certainly represent divergence events that occurred long before the formation of the Baltic Sea. These three diverse clades may have colonized the Baltic Sea at around the same time, or they may have colonised in different waves and subsequently mixed. Liu *et al. *[60] estimated that there have been 1 million years of separation between three Pacific herring (*C. pallasii*) lineages around the North American and Asian Pacific coastlines, which is of a similar order of magnitude to the dates in our study.

Multiple genetic lineages seem to be common in the Baltic region in a number of species. For example, the ninespine stickleback (*Pungitius pungitius*; [61]) apparently has only two lineages in this region, one within the Baltic and one in the North Sea. A study on the Baltic clam (*Macoma balthica*) revealed three major lineages within the Baltic Sea and North Sea, with one clade occurring only within the Baltic Sea and Alaska, a second clade occurring predominantly outside the Baltic Sea, and a third clade found in both the Baltic and North Seas; these lineages also diverged long before the formation of the Baltic Sea, at around 9.8-39 Mya [62]. It is clear that the majority of Baltic species, including herring, show a distinct barrier to gene flow between the Atlantic and the Baltic Sea [8]. This pattern indicates that Baltic Sea populations are evolutionarily divergent from the Atlantic populations, either due to isolation, bottlenecks, and/or selection for life in a brackish water environment. Perhaps surprisingly, this pattern is not evident from the distribution of mtDNA clades in our study, as the three robustly identified old lineages are found both within the Baltic and at the reference site in the Barents Sea (NO-BERLEVAG). In addition, we found no evidence for a reduction in mtDNA diversity from the entrance to the more peripheral regions within the Baltic Sea. It is clear that these lineages are now very well mixed geographically, and this may have reduced any signal of a reduction in diversity towards the innermost geographical regions, however it should be noted that out within-site sample sizes are small (5–6 individuals per site) and so diversity and differentiation measures should be considered with caution.

Similarly to our study, a whole mitochondrial genome study of the cod (*Gadus morhua*) found multiple clades but with little clear geographical patterning, despite their four sampling locations spanning the North Atlantic [26]. Although this lack of geographical structure might be expected for a wide-ranging marine species such as the cod, a recent study on Pacific herring (*C. pallasii*) did manage to detect population structure around the North American and Asian Pacific coastlines [60]. It is possible that looking at a wider geographical scale in the Atlantic herring could reveal a geographical basis for the lineages that we found, or reveal additional lineages that did not colonise the Baltic Sea.

### Isolation by distance and population structure

We did not uncover any strong patterns of isolation by distance using the whole genome, concatenated genes, or most individual genes. Our AMOVA results indicated low yet statistically significant population structuring, with an overall Φ_ST _of 0.036 for the whole genome. Although small sample sizes within-sites mean that the value of Φ_ST _may be imprecise, three sampling sites stood out as particularly distinct when looking at the amino acid data (Figure 3; DE-RUGEN, NO-BERLEVAG, and EE-MUUGA). These results strongly support findings from transcriptome-derived microsatellites in a recent study of Baltic herring [35]. Microsatellites revealed a distinct genetic unit linking DE-RUGEN, SE-STROMSTAD, DK-FREDRIKSHAVN and LV-LIEPAJA, and a separate genetic unit linking a nearby Estonian site (EE-NARVANLAHTI, which is located close to the site EE-MUUGA used in the present study) with FI-VAASA [35]. Genetic variation and differentiation in microsatellites were shown to correlate with temperature and salinity, and genetic differentiation was also found to be related to oceanographic connectivity [35]. SNP variation [34] provides additional evidence for Rügen being distinct from other Baltic populations, demonstrating introgression of North Sea herring alleles along the southern side of the Baltic, and also providing further evidence that this structure relates to adaptation to environmental heterogeneity. From our data it appears that the Rügen population (DE-RUGEN) has the lowest degree of divergence from a common ancestral population (Additional file 5), and this population is located at the root of the amino acid distance tree (Figure 3), perhaps due to introgression of North Sea haplotypes. Given the consistency of the results between the studies, it seems likely that there is some population structuring in herring within the Baltic Sea, which may have an adaptive basis.

### Evidence for selection

Although the mitochondrial genome is very conserved [63], previous studies have uncovered positive selection, particularly in the COX2 gene in high aerobic requirement fish [22], the ND2, ND4 and ND5 genes in Pacific salmon [23], and the ND2, ND4, ND5, and ATP8 genes high metabolic requirement mammals [18]. Likewise in our study, the ND2, ND4, ND5, and ATP8 genes showed the highest proportions of codons under significant positive selection. While COX2 showed one very highly significant codon, this mutation was only found in a single individual, and hence its importance can not be verified. Focussed COX2 sequencing with larger within-site sample sizes would reduce sampling variance and could provide further insight into the importance of this mutation. In contrast, a significant mutation in the ND2 gene was found at the base of clades 2 and 3, and mutations in the ND4, and ND5 genes were found at the base of clade 1. As these sites detected as likely to be under positive selection are found in the basal regions of the tree, they are likely to be important in the evolution and adaptation of Atlantic herring. The ND2, ND4 and ND5 genes together make up the transmembrane section of NADH dehydrogenase, and have been suggested to be the proton pumping device as they are homologous to a class of Na+/H+ antiporters [64]. Mutations in these three genes underlie clade 1 and clades 2/3, indicating that the clades may have some adaptive differences. In addition, the three sampling sites with the highest numbers of significant amino acid changes were the same three sites that appeared as very distinct on the amino acid multidimensional scaling plot (DE-RUGEN, NO-BERLEVAG, EE-MUUGA), providing preliminary evidence that there may be local adaptation occurring at these locations. It is also worth noting that the ATP8 gene was the only gene to show significant isolation by distance before correction for multiple testing.

The occurrence of multiple clades, and potential adaptive mutations at the base of the clades indicates that variation in the mitochondrial genome may be useful for exploring population structuring and selection at a wider global scale, in particular by providing context for the three clades found in this study. The mitochondrial genome may also be useful for detecting selection in other marine fish; according to the theory of genetic draft (a decrease in genetic diversity in regions linked to loci experiencing fixation of advantageous mutations [65]), larger populations are expected (on average) to have experienced more recent selective sweeps, resulting in reduced genetic variation relative to neutral expectations [66].

### Environmental correlates

Correlations between genetic diversity, differentiation and environmental factors were also of interest due to the strong environmental gradients in the Baltic Sea, as well as the potential adaptive value of mitochondrial variability. There was no evidence that genetic diversity (either nucleotide or amino acid) was correlated with distance from the entrance to the Baltic Sea, latitude, salinity, or temperature. However, there was some evidence that genetic distances at the COX1 gene are associated with salinity, and genetic distances at the ND3 and ND4L genes (both are members of the NADH Complex 1) are associated with temperature at spawning time, after correcting for the effects of geographical distance. ND3 genetic distance was also associated with latitude, which is likely to be due to the variation in temperature at different latitudes rather than a spatial effect, as there was no indication of isolation by distance in this gene. None of these correlations remained significant after the application of corrections for multiple testing, however, these corrections are very conservative particularly as the α values become very low given the number of tests that we have performed, and there is much debate regarding the application of such tests (for an example, see [67]). The tight linkage between the mitochondrial genes means that the tests are not truly independent, meaning that correlations of individual genes with environmental factors must be interpreted with caution.

There is very little published material on the adaptive roles of individual mitochondrial genes. However, there are indications that cytochrome oxidase enzyme activity increases in high salinity environments in the cyanobacterium *Anacystis nidulans*[68], and in the cyanobacterium *Synechococcus* 6311 cells grown in a saline environment show a tenfold increase in cytochrome oxidase activity [69]. In addition, cold stress has been shown to reduce the capacity for respiratory NADH oxidation in plants [70]. Although our results hint that the NADH complex may be important for thermal adaptation, and the Cytochrome Oxidase complex may be important for salinity adaptation, none of the genes identified as under particularly strong positive selection showed any indication of correlation with the environmental variables that we examined.

## Conclusions

From the perspective of fisheries management, this work shows that although there are distinct mitochondrial lineages of herring in the Baltic Sea, the Atlantic herring management units in the Baltic region cannot be defined on the basis of these lineages due to a lack of clear geographical structuring. However, our data does indicate higher levels of population differentiation than expected at random, with some populations appearing particularly divergent when looking at amino acid data. This divergence aligns with the within-Baltic genetic structure described using transcriptome-derived microsatellite markers [35].

There is also some indication that positive selection has occurred on the amino acids in the divergent populations. Furthermore, transcriptome-derived microsatellites showed evidence for local adaptation to temperature and selection [35], and a recent exome sequencing study [36] has provided strong evidence for positive selection in regions of the nuclear genome that are associated with adaptation to environmental heterogeneity. Together these results imply that local selection and adaptation is acting in this system. Selection on the mitochondrial genome was detected at the base of the three major lineages, and global studies covering more of the herring distribution may be able to reveal a phylogenetic pattern to these mutations.

The development of molecular markers located in selected regions of genomes has gained attention in the conservation genomics literature, the argument being that such markers may be able to play a large role in management by helping to identify management or conservation units [71]. The mitochondrial data support the units found using microsatellite markers, indicating that distinct genetic management units could be implemented. Nevertheless, filling sampling gaps on the northern coast of Poland and the Gulf of Riga (microsatellite data is missing here), and investigation into Autumn spawning herring will be critical for developing sensible management units. As for now, evidence for selection on the mitochondrial genome, and evidence for local adaptation from microsatellite markers [35] and exome sequencing [36] imply that local management of herring fisheries may be an appropriate strategy in order to maintain locally adapted populations.

## Competing interests

All authors declare that they have no competing interest.

## Authors’ contributions

AGFT carried out the molecular genetics lab work, performed all analyses except for clade dating, and wrote the paper. CWW performed the dating analysis. All authors contributed to the conception and design of the study, and contributed to drafting the final manuscript. All authors read and approved the final manuscript.

## Supplementary Material

Additional file 1 **Information on samples.** SD= fisheries subdivision, n = number of individuals. The latitude and longitude for each site are given to the accuracy provided by the collectors. Click here for file

Additional file 2 **Primer details.** Primer details including name, sequence, annealing temperature (Ta), GC content, and the length of the amplified product (in base pairs). Click here for file

Additional file 3 **Basic information and jModelTest results for whole genome and each gene.** This table shows the complex, length (in base pairs), number of segregating sites (S), model chosen by the Bayesian Information Criterion (BIC), nucleotide frequencies, substitution frequencies (R), proportion of invariable sites (p-inv), gamma shape, and transition/transversion ratio (ti/tv) for the whole genome and each gene individually. Click here for file

Additional file 4 **AMOVA results for whole genome, individual coding genes, and the control region (CR).** Presented are the Φ_ST _(a direct analogue of Wright's F_ST _for nucleotide sequence diversity), the probability that the observed data is within the range of the random data (p(rand=obs)), the probability of randomly getting an F_ST _that is higher than the observed data (P(rand>obs)), and the overall probability (P). On application of sequential Bonferroni correction, or a false discovery rate of 0.05, only the AMOVA for COX2 remains significant. Click here for file

Additional file 5 **Population-specific Φ**_**ST **_**indices from the AMOVA analyses.** Population-specific Φ_ST _indices from the AMOVA analyses, representing the degree of evolution of a particular population from a common ancestral population. These indicate whether certain populations contribute differently to others to the average Φ_ST_. Click here for file

Additional file 6 **Pairwise Φ**_**ST **_**for (a) the whole genome and (b) the COX2 gene.** Pairwise Φ_ST _for (a) the whole genome and (b) the COX2 gene. The COX2 gene showed significant population structure in the AMOVA analysis. The numbers below the diagonals are Φ_ST _and the numbers above the diagonals are the probability values. The probability values shown in bold are <0.05. Click here for file

Additional file 7 **Isolation by distance results.** The Pearson product–moment correlation coefficient statistic (r) and the probability values (P) are shown for whole genome (Genome), all genes concatenated (Genes), each individual coding gene, and the control region (CR). Separate results are shown for nucleotide and amino acid data. There is no amino acid data shown for the whole genome as large parts of the genome are non-coding, nor for the control region. In addition, amino acid results are not shown for the ND1 gene as there was no variation. Significant results are shown in bold (p<0.05). All tests were non-significant after the application of sequential Bonferroni correction or at a false discovery rate of 0.05; the corrections were applied separately for nucleotides and amino acids. Click here for file

Additional file 8 **TreeSAAP results summary.** Showing the total number of codons for each coding gene and complex, the number of these codons that were detected as being under significant positive disruptive selection (P ≤ 0.001), and the proportion of codons under selection. For each sampling site we show the number of individuals sampled, the number of codons under significant positive disruptive selection (P ≤ 0.001; only amino acid changes in terminal branches were included), and the number of codons corrected for number of individuals. Click here for file

Additional file 9 **Results of general linear models.** The response variables were the genetic diversity measures, either nucleotide diversity (nuc) or amino acid diversity (aa), while the explanatory variables were distance from the entrance to the Baltic (Distance), latitude, temperature and salinity at spawning time, temperature and salinity for April, and the interactions between temperature and salinity at spawning time and for April. F statistics (F_(1,8)_) and probability values (P) are given. P values <0.05 are shown in bold. No correlations were significant after the application of sequential Bonferroni correction or at a False Discovery Rate of 0.05; the corrections were applied within each environmental variable, and separately for nucleotides and amino acids. Click here for file

Additional file 10 **Results of Mantel and partial Mantel tests correlating genetic distance with environmental distance.** The Pearson product–moment correlation coefficient statistic (r) and the probability values (P) are shown for whole genome (Genome), all genes concatenated (Genes), each individual coding gene, and the control region (CR). Separate results are shown for nucleotide and amino acid data. There is no amino acid data shown for the whole genome as large parts of the genome are non-coding, nor for the control region. In addition, amino acid results are not shown for the ND1 gene as there was no variation. Partial Mantel tests were all performed using shortest geographical distance by sea as the conditioning variable. Significant (p<0.05) results are shown in bold. No correlations were significant after the application of sequential Bonferroni correction or at a false discovery rate of 0.05; the corrections were applied within each environmental variable, separately for nucleotides and amino acids, and separately for Mantel and partial Mantel tests. Click here for file
