# Supplementary material for: Whole mitochondrial genome scan for population structure and selection in the Atlantic herring
Source: BMC Evol Biol. 2012 Dec 22;12:248. doi: 10.1186/1471-2148-12-248 (PMC3545857; doi:10.1186/1471-2148-12-248)
Supplement: Additional file 1 — Information on samples. SD= fisheries subdivision, n = number of individuals. The latitude and longitude for each site are given to the accuracy provided by the collectors. [file 1471-2148-12-248-S1.docx]

| **Code** | **Country** | **SD** | **Latitude** | **Decimal Latitude** | **Longitude** | **Decimal Longitude** | **Collecting date** | **Approx. spawning time** | **n** |
| --- | --- | --- | --- | --- | --- | --- | --- | --- | --- |
| DE-KIEL | Germany | 22 | 54° 25' 00" N | 54.417 | 10° 2' 00" E | 10.033 | 24 March 2010 | 17 March - 31 March | 6 |
| DE-RUGEN | Germany | 24 | 54° 34' 28.28" N | 54.575 | 13° 27' 45.59" E | 13.463 | 15 April 2010 | 8 April - 22 April | 5 |
| DK-FREDRIKSHAVN | Denmark | IIIa | 57° 18' 59" N | 57.316 | 10° 32' 15" E | 10.538 | 25 April - 5 May 2010 | 18 April - 12 May | 6 |
| EE-MUDASTE | Estonia | 29 | 59^°^ 03' 6" N | 59.052 | 22^°^ 28' 3" E | 22.468 | 03 May 2009 | 26 April - 10 May | 6 |
| EE-MUUGA | Estonia | 32 | 59^°^ 32' 2" N | 59.534 | 24^°^ 51' 0" E | 24.850 | 04 May 2009 | 27 April - 11 May | 5 |
| FI-ECKERO | Finland | 29 | 60° 11' 19.34'' N | 60.189 | 19° 36' 47.87'' E | 19.613 | 19 May 2009 | 12 May -26 May | 6 |
| FI-HAUKIPUDAS | Finland | 31 | 65° 08' N | 65.133 | 24° 55' E | 24.917 | 01 June 2009 | 25 May - 8 June | 6 |
| FI-VAASA | Finland | 30 | 63° 6' 55.116" N | 63.115 | 21° 21' 10.529" E | 21.353 | 01 June 2009 | 25 May - 8 June | 6 |
| FI-VIROJOKI | Finland | 32 | 60° 30' 12" N | 60.503 | 27° 45' 30" E | 27.758 | 25 May 2009 | 18 May-1 June | 6 |
| LV-LIEPAJA | Latvia | 28-2 | 56° 47' 32.68" N | 56.792 | 21° 2' 43.83" E | 21.046 | 26 April 2010 | 19 April - 3 May | 6 |
| LV-RIGA | Latvia | 28-1 | 57° 5' 7.23" N | 57.085 | 24° 1' 4.88" E | 24.018 | 11 May 2010 | 4 May - 18 May | 6 |
| NO-BERLEVAG | Norway | Ib | 71°35'10.03" N | 71.586 | 30°25'17.26"E | 30.421 | 28 February 2008 | n/a | 5 |
| SE-BLEKINGE | Sweden | 25 | 55° 48' 9" N | 55.803 | 15° 7' 12" E | 15.120 | 05 March 2010 | 28 April - 12 May | 6 |
| SE-KALMARSUND | Sweden | 27 | 57° 23' 2" N | 57.384 | 16° 53' 2" E | 16.884 | 22 February 2010 | 15 February - 29 February | 5 |
| SE-LULEA | Sweden | 31 | 65° 25' N | 65.417 | 22° 32' E | 22.533 | 09 July 2010 | 2 July - 16 July | 6 |
| SE-STROMSTAD | Sweden | IIIa | 59° 5' 24" N | 59.090 | 11° 14' 9" E | 11.236 | 10 April 2009 | 3 April - 17 April | 6 |
| SE-UMEA | Sweden | 31 | 63° 24' 0.0'' N | 63.400 | 20° 19' 12.0'' E | 20.320 | 16 June 2009 | 9 June - 23 June | 6 |
