# Supplementary material for: Whole mitochondrial genome scan for population structure and selection in the Atlantic herring
Source: BMC Evol Biol. 2012 Dec 22;12:248. doi: 10.1186/1471-2148-12-248 (PMC3545857; doi:10.1186/1471-2148-12-248)
Supplement: Additional file 2 — Primer details. Primer details including name, sequence, annealing temperature (Ta), GC content, and the length of the amplified product (in base pairs). [file 1471-2148-12-248-S2.docx]

| **Primer Name** | **Sequence** | **Ta** | **GC%** | **Product size** |
| --- | --- | --- | --- | --- |
| CHMT1-F | GGTATTTTAACCGTGCAAAGGTAG | 60.17 | 41.67 | 974 |
| CHMT1-R | AGAGGGTCGAATGGGTTCTT | 59.93 | 50.00 |  |
| CHMT2-F | CGTCATTAATCCGCTTGCTT | 60.23 | 45.00 | 1217 |
| CHMT2-R | TTCCCAGGCCTAGGCTAGAT | 60.19 | 55.00 |  |
| CHMT3-F | TCAAAACTCTGGGTGCTTCC | 60.23 | 50.00 | 1018 |
| CHMT3-R | AAGAGCGTGAGGGACATAGC | 59.46 | 55.00 |  |
| CHMT4-F | CCCCGCTAACAGGGTTTTTA | 61.17 | 50.00 | 1094 |
| CHMT4-R | GCCAGATTTCCTGACAGAGG | 59.80 | 55.00 |  |
| CHMT5-F | TAGCCTCCTCCGGAGTTGAA | 62.17 | 55.00 | 237 |
| CHMT5-R | CTGTAACAAGAACGGATCAGACAA | 60.56 | 41.67 |  |
| CHMT6-F | CCCTCGAATAAACAATATGAGCTT | 59.90 | 37.50 | 951 |
| CHMT6-R | TTTGTTCAGGTGCTGTGGAG | 59.87 | 50.00 |  |
| CHMT7-F | TGGGAAACCCCACTTCTATG | 59.78 | 50.00 | 1164 |
| CHMT7-R | ACTCGGATCGGGGACTCTAT | 59.92 | 55.00 |  |
| CHMT8-F | TGGGACACCAATGGTACTGA | 59.81 | 50.00 | 950 |
| CHMT8-R | GGAACTGCAAGACCCATGTT | 59.97 | 50.00 |  |
| CHMT9-F | ATGCTAGGCCTTCTCCCCTA | 60.19 | 55.00 | 745 |
| CHMT9-R | CGAGTGATAAAATGCTCAGAAGAA | 59.92 | 37.50 |  |
| CHMT10-F | TCTGATTCCACTTCCACTCAATTA | 60.00 | 37.50 | 977 |
| CHMT10-R | GTAGGGCAATTTCCAGGTCA | 59.93 | 50.00 |  |
| CHMT11-F | CCTCTCCATTATCTTGGTGATTG | 59.84 | 43.48 | 1050 |
| CHMT11-R | GGGTGGCCTCAAATATCACA | 60.72 | 50.00 |  |
| CHMT12-F | CAAAACCACACTCGGACTGA | 59.72 | 50.00 | 949 |
| CHMT12-R | CGGTCAGGATGAGTGTTCAA | 59.68 | 50.00 |  |
| CHMT13-F | CTGGCAAACACCAGCTATGA | 59.86 | 50.00 | 1062 |
| CHMT13-R | TGATTCCAACACCCTCTCAA | 59.06 | 45.00 |  |
| CHMT14-F | TTCTCATCGCTATGGTCATCC | 60.05 | 47.62 | 952 |
| CHMT14-R | AGAGGTAGGAATCGGGGTGT | 59.82 | 55.00 |  |
| CHMT15-F | CGGGCTTCTTCTCCAAAGAT | 60.70 | 50.00 | 1039 |
| CHMT15-R | GCGCCTTTCTTGGCTTTAGT | 60.87 | 50.00 |  |
| CHMT16-F | CGGCTGCTAAAGCAGAAGAA | 60.78 | 50.00 | 523 |
| CHMT16-R | TAGTTTACATCTCGGCAAATGTGT | 59.96 | 37.50 |  |
| CHMT17-F | ACCCCCTTCTGAAGATTGCTAAC | 61.97 | 47.83 | 966 |
| CHMT17-R | CGGCTAGGGCTCAGAATAAA | 59.45 | 50.00 |  |
| CHMT18-F | CATACGCCATTCTTCGATCA | 59.65 | 45.00 | 1060 |
| CHMT18-R | TAGTTCACCAGGGGAGGGAAA | 62.96 | 52.38 |  |
| CHMT19-F | AACCGACCAACCAAATTGAG | 59.83 | 45.00 | 902 |
| CHMT19-R | GTGGCTGGCACGAGTTTTAC | 60.70 | 55.00 |  |
| CHMT20-F | CCCAAGGGAATTCAGCAGT | 60.06 | 52.63 | 963 |
| CHMT20-R | GGGTGGTCGAATGGTTTAGTT | 60.10 | 47.62 |  |
| CHMT21-F | GAGCCAAACAGCTAGCTCAAA | 59.78 | 47.62 | 941 |
| CHMT21-R | GAGGCGGTGTTTTTGGTAAA | 59.97 | 45.00 |  |
| CHMT22-F | CGTTAACCCCACACAGGAGT | 59.88 | 55.00 | 472 |
| CHMT22-R | GGTGTAGCTCTTGGTTTTAGGGTA | 59.96 | 45.83 |  |
