# Supplementary material for: Whole mitochondrial genome scan for population structure and selection in the Atlantic herring
Source: BMC Evol Biol. 2012 Dec 22;12:248. doi: 10.1186/1471-2148-12-248 (PMC3545857; doi:10.1186/1471-2148-12-248)
Supplement: Additional file 3 — Basic information and jModelTest results for whole genome and each gene. This table shows the complex, length (in base pairs), number of segregating sites (S), model chosen by the Bayesian Information Criterion (BIC), nucleotide frequencies, substitution frequencies (R), proportion of invariable sites (p-inv), gamma shape, and transition/transversion ratio (ti/tv) for the whole genome and each gene individually. [file 1471-2148-12-248-S3.docx]

|  | **Whole genome** | **ATP6** | **ATP8** | **COX1** | **COX2** | **COX3** | **Cytb** | **ND1** | **ND2** | **ND3** | **ND4L** | **ND4** | **ND5** | **ND6** | **CR** |
| --- | --- | --- | --- | --- | --- | --- | --- | --- | --- | --- | --- | --- | --- | --- | --- |
| Complex | NA | V | V | IV | IV | IV | III | I | I | I | I | I | I | I | NA |
| Length (bp) | 16700 | 681 | 165 | 1548 | 690 | 783 | 1140 | 972 | 1044 | 348 | 294 | 1380 | 1833 | 519 | 1047 |
| S | 1358 | 53 | 4 | 97 | 42 | 52 | 125 | 113 | 137 | 24 | 14 | 172 | 211 | 70 | 120 |
| Model | GTR+I+G | TrN+I | K80 | TrN+I | TrNef+I | K80+I | TIM3+I | TrN+I | TIM2+G | K80+I | K80+I | TrN+I+G | TIM3+I+G | TrN+G | GTR+I+G |
| freqA | 0.266 | 0.244 | NA | 0.247 | n/a | NA | 0.244 | 0.214 | 0.226 | NA | NA | 0.24 | 0.257 | 0.147 | 0.303 |
| freqC | 0.285 | 0.314 | NA | 0.264 | n/a | NA | 0.282 | 0.304 | 0.337 | NA | NA | 0.308 | 0.29 | 0.205 | 0.224 |
| freqG | 0.194 | 0.155 | NA | 0.201 | n/a | NA | 0.187 | 0.199 | 0.183 | NA | NA | 0.197 | 0.184 | 0.326 | 0.166 |
| freqT | 0.255 | 0.289 | NA | 0.289 | n/a | NA | 0.287 | 0.283 | 0.254 | NA | NA | 0.255 | 0.269 | 0.322 | 0.308 |
| R(a) [AC] | 1.106 | 1 | NA | 1 | 1 | NA | 6.256 | 1 | 0.12 | NA | NA | 1 | 10.559 | 1 | 0.965 |
| R(b) [AG] | 43.57 | 58.412 | NA | 38.947 | 32.94 | NA | 95.323 | 45.58 | 27.003 | NA | NA | 46.454 | 226.47 | 10.197 | 21.378 |
| R(c) [AT] | 0.585 | 1 | NA | 1 | 1 | NA | 1 | 1 | 0.12 | NA | NA | 1 | 1 | 1 | 0.229 |
| R(d) [CG] | 3.657 | 1 | NA | 1 | 1 | NA | 6.256 | 1 | 1 | NA | NA | 1 | 10.559 | 1 | 5.835 |
| R(e) [CT] | 13.855 | 20.455 | NA | 15.444 | 12.487 | NA | 16.003 | 9.674 | 2.739 | NA | NA | 9.284 | 47.76 | 46.953 | 10.126 |
| R(f) [GT] | 1 | 1 | NA | 1 | 1 | NA | 1 | 1 | 1 | NA | NA | 1 | 1 | 1 | 1 |
| p-inv | 0.809 | 0.808 | NA | 0.816 | 0.742 | 0.871 | 0.785 | 0.787 | n/a | 0.825 | 0.895 | 0.6 | 0.676 | n/a | 0.765 |
| gamma shape | 0.789 | n/a | NA | NA | NA | NA | NA | NA | 0.162 | NA | NA | 0.726 | 0.746 | 0.166 | 0.334 |
| ti/tv | NA | NA | 1643.208 | NA | NA | 9.444 | NA | NA | NA | 14.263 | 5043.737 | NA | NA | NA | NA |
