# Supplementary material for: Whole mitochondrial genome scan for population structure and selection in the Atlantic herring
Source: BMC Evol Biol. 2012 Dec 22;12:248. doi: 10.1186/1471-2148-12-248 (PMC3545857; doi:10.1186/1471-2148-12-248)
Supplement: Additional file 4 — AMOVA results for whole genome, individual coding genes, and the control region (CR). Presented are the ΦST (a direct analogue of Wright's FST for nucleotide sequence diversity), the probability that the observed data is within the range of the random data (p(rand=obs)), the probability of randomly getting an FST that is higher than the observed data (P(rand>obs)), and the overall probability (P). On application of sequential Bonferroni correction, or a false discovery rate of 0.05, only the AMOVA for COX2 remains significant. [file 1471-2148-12-248-S4.docx]

| **Source of variation** | **Degrees of freedom** | **Sum of squares** | **Variance components** | **Percentage of variation** | **Φ_ST_** | **P(rand>obs)** | **P(rand=obs)** | **P** |
| --- | --- | --- | --- | --- | --- | --- | --- | --- |
| **Whole genome** | | | | | | | | |
| Among populations | 16 | 968.856 | 1.844 | 3.600 |  |  |  |  |
| Within populations | 81 | 3993.633 | 49.304 | 96.400 |  |  |  |  |
| Total | 97 | 4952.490 | 51.148 | 100.000 | 0.036 | 0.022 | 0.000 | 0.022+-0.004 |
| **ATP6** | | | | | | | | |
| Among | 16 | 28.717 | 0.053 | 3.460 |  |  |  |  |
| Within | 81 | 120.467 | 1.487 | 96.540 |  |  |  |  |
| Total | 97 | 149.184 | 1.541 | 100.000 | 0.035 | 0.045 | 0.001 | 0.046+-0.002 |
| **ATP8** | | | | | | | | |
| Among | 16 | 2.133 | -0.001 | -0.870 |  |  |  |  |
| Within | 81 | 11.367 | 0.140 | 100.870 |  |  |  |  |
| Total | 97 | 13.500 | 0.139 | 100.000 | -0.009 | 0.505 | 0.028 | 0.533+-0.005 |
| **COX1** | | | | | | | | |
| Among | 16 | 51.606 | 0.089 | 3.180 |  |  |  |  |
| Within | 81 | 219.700 | 2.712 | 96.820 |  |  |  |  |
| Total | 97 | 271.306 | 2.801 | 100.000 | 0.032 | 0.084 | 0.001 | 0.085+-0.003 |
| **COX2** | | | | | | | | |
| Among | 16 | 30.525 | 0.134 | 10.530 |  |  |  |  |
| Within | 81 | 92.067 | 1.137 | 89.470 |  |  |  |  |
| Total | 97 | 122.592 | 1.270 | 100.000 | 0.105 | 0.003 | 0.000 | 0.003+-0.001 |
| **COX3** | | | | | | | | |
| Among | 16 | 38.761 | 0.072 | 3.480 |  |  |  |  |
| Within | 81 | 162.433 | 2.005 | 96.520 |  |  |  |  |
| Total | 97 | 201.194 | 2.078 | 100.000 | 0.035 | 0.050 | 0.000 | 0.050+-0.002 |
| **Cytb** | | | | | | | | |
| Among | 16 | 100.648 | 0.246 | 4.790 |  |  |  |  |
| Within | 81 | 394.933 | 4.876 | 95.210 |  |  |  |  |
| Total | 97 | 495.582 | 5.121 | 100.000 | 0.048 | 0.020 | 0.000 | 0.020+-0.001 |
| **ND1** | | | | | | | | |
| Among | 16 | 76.727 | 0.135 | 3.250 |  |  |  |  |
| Within | 81 | 325.467 | 4.018 | 96.750 |  |  |  |  |
| Total | 97 | 402.194 | 4.153 | 100.000 | 0.032 | 0.048 | 0.001 | 0.049+-0.002 |
| **ND2** | | | | | | | | |
| Among | 16 | 101.322 | 0.239 | 4.600 |  |  |  |  |
| Within | 81 | 401.433 | 4.956 | 95.400 |  |  |  |  |
| Total | 97 | 502.755 | 5.195 | 100.000 | 0.046 | 0.014 | 0.000 | 0.014+-0.001 |
| **ND3** | | | | | | | | |
| Among | 16 | 12.479 | 0.008 | 1.020 |  |  |  |  |
| Within | 81 | 59.633 | 0.736 | 98.980 |  |  |  |  |
| Total | 97 | 72.112 | 0.744 | 100.000 | 0.010 | 0.296 | 0.009 | 0.305+-0.005 |
| **ND4** | | | | | | | | |
| Among | 16 | 128.153 | 0.182 | 2.540 |  |  |  |  |
| Within | 81 | 564.000 | 6.963 | 97.460 |  |  |  |  |
| Total | 97 | 692.153 | 7.145 | 100.000 | 0.025 | 0.122 | 0.001 | 0.123+-0.003 |
| **ND4L** | | | | | | | | |
| Among | 16 | 11.816 | 0.038 | 6.790 |  |  |  |  |
| Within | 81 | 42.133 | 0.520 | 93.210 |  |  |  |  |
| Total | 97 | 53.949 | 0.558 | 100.000 | 0.068 | 0.036 | 0.002 | 0.038+-0.002 |
| **ND5** | | | | | | | | |
| Among | 16 | 151.263 | 0.292 | 3.620 |  |  |  |  |
| Within | 81 | 629.533 | 7.772 | 96.380 |  |  |  |  |
| Total | 97 | 806.388 | 8.064 | 100.000 | 0.036 | 0.031 | 0.001 | 0.032+-0.005 |
| **ND6** | | | | | | | | |
| Among | 16 | 55.705 | 0.131 | 4.580 |  |  |  |  |
| Within | 81 | 220.867 | 2.727 | 95.420 |  |  |  |  |
| Total | 97 | 276.571 | 2.858 | 100.000 | 0.046 | 0.028 | 0.001 | 0.029+-0.002 |
| **CR** | | | | | | | | |
| Among | 16 | 137.382 | 0.284 | 3.920 |  |  |  |  |
| Within | 81 | 563.067 | 6.951 | 96.080 |  |  |  |  |
| Total | 97 | 700.449 | 7.235 | 100.000 | 0.039 | 0.032 | 0.000 | 0.032+-0.005 |
