# Supplementary material for: Whole mitochondrial genome scan for population structure and selection in the Atlantic herring
Source: BMC Evol Biol. 2012 Dec 22;12:248. doi: 10.1186/1471-2148-12-248 (PMC3545857; doi:10.1186/1471-2148-12-248)
Supplement: Additional file 5 — Population-specific ΦST indices from the AMOVA analyses. Population-specific ΦST indices from the AMOVA analyses, representing the degree of evolution of a particular population from a common ancestral population. These indicate whether certain populations contribute differently to others to the average ΦST. [file 1471-2148-12-248-S5.docx]

| **Population** | **Whole genome** | **Genes** | **ATP6** | **ATP8** | **COX1** | **COX2** | **COX3** | **CYTb** | **ND1** | **ND2** | **ND3** | **ND4** | **ND4L** | **ND5** | **ND6** | **CR** |
| --- | --- | --- | --- | --- | --- | --- | --- | --- | --- | --- | --- | --- | --- | --- | --- | --- |
| DE-KIEL | 0.042 | 0.049 | -0.002 | 0.166 | 0.019 | 0.238 | -0.014 | 0.065 | 0.025 | 0.102 | -0.045 | 0.005 | 0.083 | 0.056 | 0.075 | 0.045 |
| DE-RUGEN | -0.006 | 0.000 | 0.017 | -0.196 | 0.014 | -0.043 | 0.049 | 0.000 | 0.051 | -0.002 | 0.046 | -0.043 | 0.109 | -0.011 | 0.012 | -0.029 |
| DK-FREDRIKSHAVN | 0.029 | 0.036 | 0.017 | -0.253 | 0.000 | 0.068 | 0.040 | 0.041 | 0.004 | 0.066 | 0.064 | 0.053 | 0.094 | 0.017 | 0.060 | 0.014 |
| EE-MUDASTE | 0.044 | 0.049 | 0.055 | 0.166 | 0.075 | 0.169 | 0.059 | 0.064 | 0.025 | -0.007 | 0.009 | 0.066 | -0.011 | 0.051 | 0.036 | 0.043 |
| EE-MUUGA | 0.023 | 0.023 | 0.050 | 0.166 | -0.040 | 0.128 | 0.041 | 0.011 | 0.099 | 0.005 | 0.024 | -0.010 | 0.079 | 0.020 | 0.023 | 0.071 |
| FI-ECKERO | 0.018 | 0.017 | 0.058 | -0.043 | 0.048 | 0.123 | 0.009 | 0.053 | -0.020 | 0.061 | 0.104 | -0.024 | 0.083 | -0.008 | -0.031 | 0.020 |
| FI-HAUKIPUDAS | 0.028 | 0.030 | -0.010 | -0.169 | 0.031 | 0.091 | 0.084 | 0.079 | -0.019 | 0.007 | 0.002 | 0.043 | 0.146 | 0.008 | 0.024 | 0.031 |
| FI-VAASA | 0.015 | 0.019 | 0.020 | 0.166 | -0.006 | 0.192 | -0.081 | 0.033 | 0.040 | 0.045 | -0.100 | -0.008 | 0.042 | 0.015 | 0.034 | 0.019 |
| FI-VIROJOKI | 0.037 | 0.040 | 0.096 | -0.169 | 0.037 | 0.008 | 0.009 | 0.040 | 0.000 | 0.057 | -0.053 | 0.040 | 0.177 | 0.056 | 0.036 | 0.029 |
| LV-LIEPAJA | 0.098 | 0.103 | 0.108 | -0.043 | 0.077 | 0.146 | 0.107 | 0.119 | 0.054 | 0.104 | 0.143 | 0.126 | -0.011 | 0.110 | 0.081 | 0.089 |
| LV-RIGA | 0.020 | 0.027 | 0.017 | 0.166 | 0.035 | 0.105 | -0.019 | 0.022 | 0.054 | 0.052 | 0.104 | -0.050 | 0.083 | 0.032 | 0.085 | 0.011 |
| NO-BERLEVAG | 0.018 | 0.017 | -0.016 | 0.166 | -0.040 | 0.128 | 0.081 | -0.002 | 0.067 | 0.003 | 0.046 | -0.017 | 0.079 | 0.014 | 0.041 | 0.039 |
| SE-BLEKINGE | 0.096 | 0.099 | 0.126 | -0.043 | 0.108 | 0.155 | 0.090 | 0.074 | 0.068 | 0.099 | 0.025 | 0.115 | 0.230 | 0.101 | 0.095 | 0.103 |
| SE-KALMARSUND | 0.049 | 0.048 | 0.028 | 0.166 | 0.008 | 0.023 | 0.081 | 0.075 | 0.051 | 0.043 | 0.001 | 0.049 | -0.011 | 0.040 | 0.076 | 0.055 |
| SE-LULEA | 0.023 | 0.021 | 0.009 | -0.253 | 0.062 | 0.146 | 0.020 | -0.001 | 0.022 | 0.050 | -0.100 | 0.004 | -0.042 | 0.011 | 0.038 | 0.054 |
| SE-STROMSTAD | 0.033 | 0.035 | -0.033 | -0.043 | 0.029 | 0.077 | 0.062 | 0.055 | 0.040 | 0.018 | 0.072 | 0.015 | -0.011 | 0.047 | 0.046 | 0.022 |
| SE-UMEA | 0.037 | 0.038 | 0.039 | -0.043 | 0.052 | 0.008 | -0.005 | 0.069 | 0.014 | 0.057 | -0.155 | 0.047 | 0.031 | 0.041 | 0.044 | 0.047 |
| All populations | 0.036 | 0.039 | 0.035 | -0.009 | 0.032 | 0.105 | 0.035 | 0.048 | 0.032 | 0.046 | 0.010 | 0.025 | 0.068 | 0.036 | 0.046 | 0.039 |
