# Supplementary material for: Whole mitochondrial genome scan for population structure and selection in the Atlantic herring
Source: BMC Evol Biol. 2012 Dec 22;12:248. doi: 10.1186/1471-2148-12-248 (PMC3545857; doi:10.1186/1471-2148-12-248)
Supplement: Additional file 6 — Pairwise ΦST for (a) the whole genome and (b) the COX2 gene. Pairwise ΦST for (a) the whole genome and (b) the COX2 gene. The COX2 gene showed significant population structure in the AMOVA analysis. The numbers below the diagonals are ΦST and the numbers above the diagonals are the probability values. The probability values shown in bold are <0.05. [file 1471-2148-12-248-S6.docx]

1. Whole genome

|  | DE-KIEL | DE-RUGEN | DK-FREDRIKSHAVN | EE-MUDASTE | EE-MUUGA | FI-ECKERO | FI-HAUKIPUDAS | FI-VAASA | FI-VIROJOKI | LV-LIEPAJA | LV-RIGA | NO-BERLEVAG | SE-BLEKINGE | SE-KALMARSUND | SE-LULEA | SE-STROMSTAD | SE-UMEA |
| --- | --- | --- | --- | --- | --- | --- | --- | --- | --- | --- | --- | --- | --- | --- | --- | --- | --- |
| DE-KIEL |  | 0.090 | **0.027** | 0.207 | 0.450 | 0.243 | **0.009** | 0.865 | **0.000** | **0.027** | 0.171 | 0.712 | **0.000** | 0.297 | 0.838 | 0.081 | 0.423 |
| DE-RUGEN | 0.100 |  | 0.324 | **0.027** | 0.649 | 0.523 | 0.496 | 0.189 | 0.865 | 0.712 | 0.432 | 0.640 | **0.018** | 0.144 | 0.396 | 0.225 | 0.171 |
| DK-FREDRIKSHAVN | 0.095 | -0.011 |  | 0.198 | 0.775 | 0.676 | 0.712 | 0.072 | 0.649 | 0.829 | 0.288 | 0.595 | 0.063 | 0.550 | 0.550 | 0.676 | 0.811 |
| EE-MUDASTE | 0.039 | 0.134 | 0.043 |  | 0.577 | 0.225 | 0.081 | 0.225 | **0.045** | **0.027** | **0.027** | 0.459 | **0.000** | 0.739 | 0.991 | 0.342 | 0.568 |
| EE-MUUGA | -0.034 | -0.026 | -0.041 | -0.009 |  | 0.820 | 0.369 | 0.703 | 0.117 | 0.351 | 0.712 | 0.973 | **0.000** | 0.991 | 0.910 | 0.838 | 0.910 |
| FI-ECKERO | 0.002 | -0.025 | -0.028 | 0.015 | -0.076 |  | 0.432 | 0.297 | 0.523 | 0.604 | 0.748 | 0.928 | **0.018** | 0.847 | 0.910 | 0.847 | 0.973 |
| FI-HAUKIPUDAS | 0.129 | -0.013 | -0.048 | 0.073 | 0.002 | -0.015 |  | **0.036** | 0.523 | 0.405 | 0.333 | 0.306 | **0.009** | 0.243 | 0.405 | 0.532 | 0.270 |
| FI-VAASA | -0.103 | 0.072 | 0.086 | 0.046 | -0.058 | -0.014 | 0.108 |  | **0.009** | **0.018** | 0.622 | 0.784 | **0.000** | 0.396 | 0.910 | 0.261 | 0.432 |
| FI-VIROJOKI | 0.187 | -0.050 | -0.035 | 0.144 | 0.043 | -0.011 | -0.036 | 0.168 |  | 0.486 | 0.279 | 0.180 | 0.225 | 0.162 | 0.144 | 0.342 | 0.225 |
| LV-LIEPAJA | 0.154 | -0.027 | -0.049 | 0.125 | 0.006 | -0.025 | -0.012 | 0.130 | -0.033 |  | 0.414 | 0.324 | **0.018** | 0.270 | 0.153 | 0.351 | 0.441 |
| LV-RIGA | 0.022 | -0.041 | 0.029 | 0.111 | -0.076 | -0.061 | 0.031 | -0.035 | 0.037 | 0.033 |  | 0.649 | **0.009** | 0.234 | 0.342 | 0.360 | 0.270 |
| NO-BERLEVAG | -0.052 | -0.025 | -0.023 | 0.000 | -0.158 | -0.072 | 0.011 | -0.067 | 0.048 | 0.004 | -0.063 |  | **0.000** | 0.991 | 0.829 | 0.712 | 0.928 |
| SE-BLEKINGE | 0.394 | 0.095 | 0.119 | 0.383 | 0.263 | 0.205 | 0.152 | 0.360 | 0.042 | 0.153 | 0.216 | 0.258 |  | **0.000** | **0.000** | **0.000** | **0.000** |
| SE-KALMARSUND | 0.016 | 0.035 | -0.024 | -0.026 | -0.072 | -0.034 | 0.022 | 0.031 | 0.059 | 0.026 | 0.051 | -0.073 | 0.303 |  | 0.982 | 0.802 | 0.973 |
| SE-LULEA | -0.072 | 0.019 | -0.017 | -0.051 | -0.098 | -0.070 | 0.006 | -0.074 | 0.054 | 0.027 | -0.021 | -0.094 | 0.285 | -0.067 |  | 0.946 | 0.973 |
| SE-STROMSTAD | 0.062 | 0.015 | -0.041 | 0.001 | -0.039 | -0.037 | -0.033 | 0.055 | 0.005 | -0.008 | 0.031 | -0.031 | 0.232 | -0.036 | -0.056 |  | 0.838 |
| SE-UMEA | 0.016 | 0.033 | -0.020 | -0.012 | -0.040 | -0.046 | 0.006 | 0.017 | 0.039 | -0.002 | 0.041 | -0.042 | 0.273 | -0.057 | -0.071 | -0.045 |  |

(b) COX2

|  | DE-KIEL | DE-RUGEN | DK-FREDRIKSHAVN | EE-MUDASTE | EE-MUUGA | FI-ECKERO | FI-HAUKIPUDAS | FI-VAASA | FI-VIROJOKI | LV-LIEPAJA | LV-RIGA | NO-BERLEVAG | SE-BLEKINGE | SE-KALMARSUND | SE-LULEA | SE-STROMSTAD | SE-UMEA |
| --- | --- | --- | --- | --- | --- | --- | --- | --- | --- | --- | --- | --- | --- | --- | --- | --- | --- |
| DE-KIEL |  | **0.036** | 0.207 | 0.991 | 0.369 | 0.991 | 0.180 | 0.991 | 0.072 | 0.216 | 0.532 | 0.243 | **0.000** | 0.063 | 0.991 | 0.243 | 0.991 |
| DE-RUGEN | 0.259 |  | 0.892 | **0.036** | 0.757 | 0.198 | 0.757 | **0.018** | 0.919 | 0.613 | 0.450 | 0.658 | **0.045** | 0.144 | 0.180 | 0.829 | 0.225 |
| DK-FREDRIKSHAVN | 0.277 | -0.080 |  | 0.171 | 0.315 | 0.532 | 0.991 | 0.189 | 0.991 | 0.991 | 0.991 | 0.333 | 0.180 | 0.153 | 0.586 | 0.991 | 0.577 |
| EE-MUDASTE | 0.000 | 0.168 | 0.173 |  | 0.739 | 0.991 | 0.117 | 0.991 | 0.108 | 0.252 | 0.423 | 0.658 | **0.000** | 0.486 | 0.991 | 0.306 | 0.991 |
| EE-MUUGA | 0.028 | 0.018 | 0.070 | -0.034 |  | 0.820 | 0.270 | 0.234 | 0.225 | 0.405 | 0.423 | 0.991 | **0.000** | 0.991 | 0.883 | 0.405 | 0.973 |
| FI-ECKERO | 0.000 | 0.064 | 0.018 | -0.034 | -0.071 |  | 0.468 | 0.991 | 0.171 | 0.496 | 0.991 | 0.829 | **0.018** | 0.604 | 0.991 | 0.477 | 0.991 |
| FI-HAUKIPUDAS | 0.300 | -0.083 | -0.129 | 0.186 | 0.078 | 0.043 |  | 0.162 | 0.991 | 0.991 | 0.991 | 0.297 | 0.081 | 0.108 | 0.577 | 0.991 | 0.559 |
| FI-VAASA | 0.000 | 0.228 | 0.240 | 0.000 | 0.013 | -0.038 | 0.257 |  | 0.072 | 0.189 | 0.414 | 0.333 | **0.000** | 0.189 | 0.991 | 0.180 | 0.991 |
| FI-VIROJOKI | 0.333 | -0.134 | -0.078 | 0.237 | 0.094 | 0.121 | -0.082 | 0.300 |  | 0.658 | 0.532 | 0.216 | 0.171 | 0.099 | 0.270 | 0.775 | 0.189 |
| LV-LIEPAJA | 0.250 | -0.064 | -0.117 | 0.100 | 0.003 | -0.031 | -0.127 | 0.200 | -0.043 |  | 0.991 | 0.252 | **0.009** | 0.126 | 0.477 | 0.991 | 0.523 |
| LV-RIGA | 0.133 | -0.039 | -0.086 | 0.047 | -0.034 | -0.067 | -0.092 | 0.109 | -0.008 | -0.142 |  | 0.378 | **0.018** | 0.450 | 0.991 | 0.991 | 0.991 |
| NO-BERLEVAG | 0.028 | 0.018 | 0.070 | -0.034 | -0.190 | -0.071 | 0.078 | 0.013 | 0.094 | 0.003 | -0.034 |  | **0.000** | 0.991 | 0.820 | 0.432 | 0.982 |
| SE-BLEKINGE | 0.767 | 0.177 | 0.204 | 0.656 | 0.567 | 0.525 | 0.250 | 0.708 | 0.117 | 0.400 | 0.400 | 0.567 |  | **0.009** | **0.009** | **0.009** | **0.027** |
| SE-KALMARSUND | 0.033 | 0.068 | 0.116 | -0.011 | -0.094 | -0.014 | 0.124 | 0.022 | 0.128 | 0.085 | 0.009 | -0.094 | 0.518 |  | 0.450 | 0.342 | 0.838 |
| SE-LULEA | 0.000 | 0.049 | 0.043 | -0.038 | -0.120 | -0.100 | 0.046 | 0.000 | 0.111 | -0.034 | -0.073 | -0.120 | 0.564 | -0.040 |  | 0.631 | 0.991 |
| SE-STROMSTAD | 0.182 | -0.081 | -0.093 | 0.100 | -0.005 | -0.024 | -0.100 | 0.154 | -0.056 | -0.114 | -0.138 | -0.005 | 0.337 | 0.027 | -0.026 |  | 0.901 |
| SE-UMEA | 0.000 | 0.043 | 0.030 | -0.023 | -0.059 | -0.063 | 0.032 | 0.000 | 0.096 | -0.021 | -0.068 | -0.059 | 0.429 | -0.038 | -0.067 | -0.056 |  |
