# Supplementary material for: Whole mitochondrial genome scan for population structure and selection in the Atlantic herring
Source: BMC Evol Biol. 2012 Dec 22;12:248. doi: 10.1186/1471-2148-12-248 (PMC3545857; doi:10.1186/1471-2148-12-248)
Supplement: Additional file 7 — Isolation by distance results. The Pearson product–moment correlation coefficient statistic (r) and the probability values (P) are shown for whole genome (Genome), all genes concatenated (Genes), each individual coding gene, and the control region (CR). Separate results are shown for nucleotide and amino acid data. There is no amino acid data shown for the whole genome as large parts of the genome are non-coding, nor for the control region. In addition, amino acid results are not shown for the ND1 gene as there was no variation. Significant results are shown in bold (p<0.05). All tests were non-significant after the application of sequential Bonferroni correction or at a false discovery rate of 0.05; the corrections were applied separately for nucleotides and amino acids. [file 1471-2148-12-248-S7.docx]

|  | **Nucleotides** | | **Amino Acids** | |
| --- | --- | --- | --- | --- |
|  | **r** | **P** | **r** | **P** |
| Genome | 0.013 | 0.467 | NA | NA |
| Genes | -0.002 | 0.519 | -0.128 | 0.816 |
| ATP6 | 0.210 | 0.064 | 0.162 | 0.150 |
| ATP8 | 0.282 | **0.030** | 0.271 | 0.060 |
| COX1 | -0.029 | 0.576 | 0.116 | 0.236 |
| COX2 | -0.055 | 0.639 | 0.115 | 0.221 |
| COX3 | 0.008 | 0.470 | -0.013 | 0.501 |
| Cytb | -0.010 | 0.533 | 0.012 | 0.468 |
| ND1 | 0.050 | 0.366 | NA | NA |
| ND2 | -0.043 | 0.624 | -0.121 | 0.771 |
| ND3 | 0.109 | 0.236 | 0.070 | 0.318 |
| ND4L | -0.019 | 0.548 | -0.011 | 0.440 |
| ND4 | 0.008 | 0.479 | 0.026 | 0.433 |
| ND5 | -0.005 | 0.527 | -0.092 | 0.718 |
| ND6 | -0.146 | 0.869 | 0.074 | 0.309 |
