# Supplementary material for: Whole mitochondrial genome scan for population structure and selection in the Atlantic herring
Source: BMC Evol Biol. 2012 Dec 22;12:248. doi: 10.1186/1471-2148-12-248 (PMC3545857; doi:10.1186/1471-2148-12-248)
Supplement: Additional file 8 — TreeSAAP results summary. Showing the total number of codons for each coding gene and complex, the number of these codons that were detected as being under significant positive disruptive selection (P ≤ 0.001), and the proportion of codons under selection. For each sampling site we show the number of individuals sampled, the number of codons under significant positive disruptive selection (P ≤ 0.001; only amino acid changes in terminal branches were included), and the number of codons corrected for number of individuals. [file 1471-2148-12-248-S8.docx]

| **Gene** | **No. Codons** | **No. Codons under selection** | **Proportion of codons under selection** |
| --- | --- | --- | --- |
| ND2 | 347 | 17 | 0.049 |
| ND5 | 611 | 28 | 0.046 |
| ND4 | 459 | 20 | 0.044 |
| ATP8 | 55 | 2 | 0.036 |
| ATP6 | 227 | 4 | 0.018 |
| ND3 | 116 | 2 | 0.017 |
| ND4L | 98 | 1 | 0.010 |
| COX2 | 229 | 2 | 0.009 |
| CytB | 379 | 3 | 0.008 |
| COX3 | 261 | 1 | 0.004 |
| COX1 | 516 | 1 | 0.002 |
| ND1 | 325 | 0 | 0.000 |
| ND6 | 173 | 0 | 0.000 |
| **Complex** | **No. Codons** | **No. Codons under selection** | **Proportion of codons under selection** |
| ND | 2129 | 68 | 0.032 |
| ATP | 282 | 6 | 0.021 |
| Cytb | 379 | 3 | 0.008 |
| COX | 1006 | 4 | 0.004 |
| **Site** | **No. Individuals** | **No. Codons under selection** | **No. Codons under selection/No. Individuals** |
| DE-RUGEN | 5 | 7 | 1.400 |
| EE-MUUGA | 5 | 7 | 1.400 |
| NO-BERLEVAG | 5 | 6 | 1.200 |
| DE-KIEL | 6 | 5 | 0.833 |
| DK-FREDRIKSHAVN | 6 | 5 | 0.833 |
| EE-MUDASTE | 6 | 5 | 0.833 |
| FI-HAUKIPUDAS | 6 | 5 | 0.833 |
| SE-STROMSTAD | 6 | 5 | 0.833 |
| SE-KALMARSUND | 5 | 4 | 0.800 |
| FI-VIROJOKI | 6 | 4 | 0.667 |
| LV-RIGA | 6 | 4 | 0.667 |
| SE-BLEKINGE | 6 | 4 | 0.667 |
| SE-UMEA | 6 | 4 | 0.667 |
| FI-ECKERO | 6 | 3 | 0.500 |
| SE-LULEA | 6 | 3 | 0.500 |
| FI-VAASA | 6 | 2 | 0.333 |
| LV-LIEPAJA | 6 | 0 | 0.000 |
