# Supplementary material for: Whole mitochondrial genome scan for population structure and selection in the Atlantic herring
Source: BMC Evol Biol. 2012 Dec 22;12:248. doi: 10.1186/1471-2148-12-248 (PMC3545857; doi:10.1186/1471-2148-12-248)
Supplement: Additional file 9 — Results of general linear models. The response variables were the genetic diversity measures, either nucleotide diversity (nuc) or amino acid diversity (aa), while the explanatory variables were distance from the entrance to the Baltic (Distance), latitude, temperature and salinity at spawning time, temperature and salinity for April, and the interactions between temperature and salinity at spawning time and for April. F statistics (F(1,8)) and probability values (P) are given. P values <0.05 are shown in bold. No correlations were significant after the application of sequential Bonferroni correction or at a False Discovery Rate of 0.05; the corrections were applied within each environmental variable, and separately for nucleotides and amino acids. [file 1471-2148-12-248-S9.docx]

|  | | **Distance** | | **Latitude** | | **Spawning temp** | | **Spawning salinity** | | **April temp** | | **April salinity** | | **Spawning temp:salinity** | | **April temp:salinity** | |
| --- | --- | --- | --- | --- | --- | --- | --- | --- | --- | --- | --- | --- | --- | --- | --- | --- | --- |
|  |  | **F_(1,8)_** | **P** | **F_(1,8)_** | **P** | **F_(1,8)_** | **P** | **F_(1,8)_** | **P** | **F_(1,8)_** | **P** | **F_(1,8)_** | **P** | **F_(1,8)_** | **P** | **F_(1,8)_** | **P** |
| **Genome** | **nuc** | 0.039 | 0.848 | 0.017 | 0.900 | 0.162 | 0.700 | 0.267 | 0.621 | 3.286 | 0.113 | 0.018 | 0.898 | 0.004 | 0.950 | 0.004 | 0.949 |
| **Genes** | **nuc** | 0.060 | 0.814 | 0.006 | 0.943 | 0.131 | 0.728 | 0.155 | 0.705 | 3.158 | 0.119 | 0.007 | 0.937 | 0.000 | 1.000 | 0.026 | 0.874 |
|  | **aa** | 0.328 | 0.585 | 0.162 | 0.700 | 0.352 | 0.572 | 0.292 | 0.605 | 4.435 | 0.073 | 0.185 | 0.680 | 0.108 | 0.748 | 0.288 | 0.601 |
| **APT6** | **nuc** | 0.020 | 0.892 | 0.140 | 0.720 | 0.675 | 0.438 | 0.079 | 0.787 | 0.371 | 0.562 | 0.423 | 0.536 | 1.077 | 0.318 | 1.956 | 0.185 |
|  | **aa** | 0.207 | 0.663 | 6.349 | **0.040** | 5.541 | 0.051 | 0.080 | 0.785 | 1.421 | 0.272 | 0.003 | 0.955 | 0.475 | 0.503 | 0.014 | 0.909 |
| **ATP8** | **nuc** | 1.702 | 0.233 | 0.733 | 0.420 | 6.913 | **0.034** | 0.002 | 0.968 | 0.620 | 0.457 | 0.066 | 0.805 | 5.866 | **0.031** | 3.837 | 0.072 |
|  | **aa** | 2.077 | 0.193 | 2.526 | 0.156 | 0.307 | 0.597 | 0.001 | 0.982 | 0.657 | 0.444 | 1.221 | 0.306 | 2.566 | 0.133 | 0.905 | 0.359 |
| **COX1** | **nuc** | 0.427 | 0.534 | 0.018 | 0.897 | 3.695 | 0.096 | 0.109 | 0.751 | 0.120 | 0.739 | 0.461 | 0.519 | 0.802 | 0.387 | 1.056 | 0.323 |
|  | **aa** | 0.031 | 0.865 | 0.069 | 0.800 | 0.832 | 0.392 | 0.726 | 0.422 | 0.006 | 0.942 | 0.534 | 0.489 | 0.086 | 0.774 | 0.366 | 0.556 |
| **COX2** | **nuc** | 0.134 | 0.726 | 0.116 | 0.743 | 1.341 | 0.285 | 1.223 | 0.305 | 1.252 | 0.300 | 0.774 | 0.408 | 0.600 | 0.453 | 0.551 | 0.471 |
|  | **aa** | 0.571 | 0.474 | 1.327 | 0.287 | 0.432 | 0.532 | 0.228 | 0.647 | 0.359 | 0.568 | 0.014 | 0.909 | 1.262 | 0.282 | 1.751 | 0.209 |
| **COX3** | **nuc** | 0.012 | 0.915 | 0.088 | 0.776 | 0.450 | 0.524 | 3.242 | 0.115 | 0.829 | 0.393 | 1.945 | 0.206 | 0.032 | 0.860 | 0.160 | 0.696 |
|  | **aa** | 0.461 | 0.519 | 0.249 | 0.633 | 5.805 | **0.047** | 0.473 | 0.514 | 0.880 | 0.379 | 0.170 | 0.693 | 0.784 | 0.392 | 1.104 | 0.313 |
| **Cytb** | **nuc** | 0.252 | 0.631 | 0.225 | 0.649 | 0.596 | 0.465 | 0.405 | 0.545 | 3.895 | 0.089 | 0.198 | 0.670 | 0.069 | 0.797 | 0.152 | 0.703 |
|  | **aa** | 0.073 | 0.795 | 0.091 | 0.771 | 0.500 | 0.503 | 0.524 | 0.493 | 3.996 | 0.086 | 0.046 | 0.837 | 0.057 | 0.815 | 0.474 | 0.503 |
| **ND1** | **nuc** | 0.008 | 0.932 | 0.021 | 0.889 | 0.143 | 0.717 | 0.037 | 0.853 | 0.526 | 0.492 | 0.043 | 0.841 | 1.842 | 0.198 | 1.575 | 0.214 |
|  | **aa** | NA | NA | NA | NA | NA | NA | NA | NA | NA | NA | NA | NA | NA | NA | NA | NA |
| **ND2** | **nuc** | 0.055 | 0.821 | 0.079 | 0.786 | 0.893 | 0.376 | 0.423 | 0.536 | 3.313 | 0.112 | 0.950 | 0.362 | 0.053 | 0.821 | 0.000 | 0.999 |
|  | **aa** | 0.698 | 0.431 | 0.005 | 0.946 | 0.454 | 0.522 | 2.580 | 0.152 | 5.025 | 0.060 | 1.528 | 0.256 | 0.438 | 0.520 | 0.653 | 0.434 |
| **ND3** | **nuc** | 1.346 | 0.284 | 0.994 | 0.352 | 0.149 | 0.711 | 0.141 | 0.718 | 0.770 | 0.409 | 0.463 | 0.518 | 0.145 | 0.710 | 0.656 | 0.433 |
|  | **aa** | 1.412 | 0.273 | 2.616 | 0.150 | 0.169 | 0.693 | 0.407 | 0.544 | 3.596 | 0.100 | 0.025 | 0.878 | 0.370 | 0.553 | 0.083 | 0.777 |
| **ND4L** | **nuc** | 0.323 | 0.587 | 0.444 | 0.527 | 0.005 | 0.944 | 3.349 | 0.110 | 0.166 | 0.696 | 3.294 | 0.112 | 0.160 | 0.696 | 0.210 | 0.655 |
|  | **aa** | 3.235 | 0.115 | 0.685 | 0.435 | 0.280 | 0.613 | 0.118 | 0.742 | 1.484 | 0.263 | 0.809 | 0.398 | 0.902 | 0.360 | 0.727 | 0.409 |
| **ND4** | **nuc** | 0.009 | 0.927 | 0.616 | 0.458 | 0.174 | 0.689 | 0.772 | 0.409 | 1.846 | 0.216 | 0.316 | 0.592 | 0.611 | 0.448 | 0.324 | 0.579 |
|  | **aa** | 0.197 | 0.671 | 1.564 | 0.251 | 0.091 | 0.772 | 0.477 | 0.512 | 4.466 | 0.072 | 0.005 | 0.944 | 0.576 | 0.461 | 1.085 | 0.317 |
| **ND5** | **nuc** | 0.286 | 0.610 | 0.196 | 0.671 | 0.187 | 0.679 | 0.007 | 0.936 | 2.033 | 0.197 | 0.007 | 0.936 | 0.086 | 0.774 | 0.187 | 0.673 |
|  | **aa** | 0.065 | 0.807 | 0.910 | 0.372 | 0.257 | 0.628 | 0.939 | 0.365 | 2.800 | 0.138 | 0.719 | 0.425 | 0.066 | 0.801 | 0.448 | 0.515 |
| **ND6** | **nuc** | 0.960 | 0.360 | 1.314 | 0.289 | 0.000 | 0.992 | 3.089 | 0.122 | 3.213 | 0.116 | 3.404 | 0.108 | 0.719 | 0.418 | 1.741 | 0.210 |
|  | **aa** | 1.871 | 0.214 | 2.692 | 0.145 | 0.131 | 0.729 | 4.989 | 0.061 | 0.003 | 0.955 | 4.712 | 0.067 | 0.455 | 0.512 | 0.123 | 0.731 |
| **CR** | **nuc** | 0.003 | 0.959 | 0.125 | 0.734 | 0.269 | 0.620 | 0.241 | 0.639 | 2.695 | 0.145 | 0.003 | 0.960 | 0.126 | 0.728 | 0.189 | 0.671 |
