# Supplementary material for: Whole mitochondrial genome scan for population structure and selection in the Atlantic herring
Source: BMC Evol Biol. 2012 Dec 22;12:248. doi: 10.1186/1471-2148-12-248 (PMC3545857; doi:10.1186/1471-2148-12-248)
Supplement: Additional file 10 — Results of Mantel and partial Mantel tests correlating genetic distance with environmental distance. The Pearson product–moment correlation coefficient statistic (r) and the probability values (P) are shown for whole genome (Genome), all genes concatenated (Genes), each individual coding gene, and the control region (CR). Separate results are shown for nucleotide and amino acid data. There is no amino acid data shown for the whole genome as large parts of the genome are non-coding, nor for the control region. In addition, amino acid results are not shown for the ND1 gene as there was no variation. Partial Mantel tests were all performed using shortest geographical distance by sea as the conditioning variable. Significant (p<0.05) results are shown in bold. No correlations were significant after the application of sequential Bonferroni correction or at a false discovery rate of 0.05; the corrections were applied within each environmental variable, separately for nucleotides and amino acids, and separately for Mantel and partial Mantel tests. [file 1471-2148-12-248-S10.docx]

| **x** | **y** | **Nucleotides** | | | | **Amino Acids** | | | |
| --- | --- | --- | --- | --- | --- | --- | --- | --- | --- |
|  |  | **Mantel** | | **Partial Mantel** | | **Mantel** | | **Partial Mantel** | |
|  |  | **r** | **P** | **r** | **P** | **r** | **P** | **r** | **P** |
| Genome | April salinity | -0.074 | 0.734 | -0.110 | 0.783 | NA | NA | NA | NA |
| Genes | April salinity | -0.103 | 0.818 | -0.136 | 0.830 | -0.129 | 0.744 | -0.059 | 0.598 |
| ATP6 | April salinity | 0.299 | 0.057 | 0.218 | 0.134 | 0.145 | 0.299 | 0.051 | 0.401 |
| ATP8 | April salinity | 0.268 | 0.126 | 0.113 | 0.301 | 0.404 | **0.035** | 0.311 | 0.156 |
| COX1 | April salinity | 0.034 | 0.433 | 0.071 | 0.372 | 0.385 | **0.039** | 0.414 | **0.033** |
| COX2 | April salinity | -0.091 | 0.621 | -0.073 | 0.600 | 0.361 | 0.096 | 0.383 | 0.101 |
| COX3 | April salinity | -0.044 | 0.546 | -0.065 | 0.597 | -0.079 | 0.376 | -0.094 | 0.690 |
| Cytb | April salinity | -0.111 | 0.721 | -0.139 | 0.769 | 0.169 | 0.239 | 0.216 | 0.175 |
| ND1 | April salinity | -0.003 | 0.538 | -0.048 | 0.616 | NA | NA | NA | NA |
| ND2 | April salinity | -0.145 | 0.753 | -0.156 | 0.777 | -0.314 | 0.944 | -0.315 | 0.949 |
| ND3 | April salinity | -0.158 | 0.737 | -0.310 | 0.927 | 0.174 | 0.215 | 0.171 | 0.246 |
| ND4 | April salinity | -0.084 | 0.636 | -0.119 | 0.721 | 0.052 | 0.393 | 0.047 | 0.403 |
| ND4L | April salinity | 0.051 | 0.372 | 0.086 | 0.328 | -0.094 | 0.571 | -0.116 | 0.815 |
| ND5 | April salinity | -0.086 | 0.734 | -0.111 | 0.780 | -0.293 | 0.910 | -0.310 | 0.931 |
| ND6 | April salinity | -0.257 | 0.913 | -0.216 | 0.885 | -0.168 | 0.616 | -0.290 | 0.969 |
| CR | April salinity | 0.021 | 0.409 | 0.002 | 0.467 | NA | NA | NA | NA |
| Genome | April temp | -0.230 | 0.958 | -0.305 | 0.948 | NA | NA | NA | NA |
| Genes | April temp | -0.254 | 0.975 | -0.324 | 0.950 | -0.193 | 0.936 | -0.146 | 0.838 |
| ATP6 | April temp | -0.080 | 0.727 | -0.276 | 0.940 | 0.039 | 0.383 | -0.081 | 0.600 |
| ATP8 | April temp | 0.154 | 0.140 | -0.029 | 0.505 | 0.174 | 0.138 | 0.007 | 0.325 |
| COX1 | April temp | -0.134 | 0.846 | -0.148 | 0.820 | 0.205 | 0.090 | 0.171 | 0.186 |
| COX2 | April temp | -0.131 | 0.846 | -0.124 | 0.759 | 0.066 | 0.333 | -0.008 | 0.360 |
| COX3 | April temp | -0.098 | 0.753 | -0.132 | 0.786 | 0.015 | 0.312 | 0.029 | 0.309 |
| Cytb | April temp | -0.191 | 0.926 | -0.236 | 0.935 | -0.146 | 0.838 | -0.196 | 0.866 |
| ND1 | April temp | 0.019 | 0.456 | -0.016 | 0.557 | NA | NA | NA | NA |
| ND2 | April temp | -0.239 | 0.969 | -0.271 | 0.965 | -0.242 | 0.975 | -0.215 | 0.926 |
| ND3 | April temp | -0.142 | 0.834 | -0.271 | 0.957 | 0.238 | 0.060 | 0.249 | 0.142 |
| ND4 | April temp | -0.263 | 0.982 | -0.342 | 0.977 | -0.219 | 0.947 | -0.301 | 0.979 |
| ND4L | April temp | 0.094 | 0.225 | 0.135 | 0.207 | -0.087 | 0.686 | -0.102 | 0.681 |
| ND5 | April temp | -0.269 | 0.976 | -0.340 | 0.954 | -0.294 | 0.992 | -0.304 | 0.985 |
| ND6 | April temp | -0.234 | 0.980 | -0.185 | 0.902 | -0.129 | 0.800 | -0.225 | 0.986 |
| CR | April temp | -0.123 | 0.834 | -0.180 | 0.901 | NA | NA | NA | NA |
| Genome | Spawning salinity | -0.087 | 0.759 | -0.123 | 0.793 | NA | NA | NA | NA |
| Genes | Spawning salinity | -0.114 | 0.827 | -0.146 | 0.828 | -0.141 | 0.759 | -0.078 | 0.644 |
| ATP6 | Spawning salinity | 0.312 | **0.047** | 0.236 | 0.110 | 0.143 | 0.286 | 0.052 | 0.399 |
| ATP8 | Spawning salinity | 0.231 | 0.162 | 0.071 | 0.371 | 0.381 | **0.043** | 0.281 | 0.168 |
| COX1 | Spawning salinity | 0.046 | 0.408 | 0.083 | 0.339 | 0.387 | **0.039** | 0.408 | **0.041** |
| COX2 | Spawning salinity | -0.076 | 0.604 | -0.053 | 0.576 | 0.337 | 0.139 | 0.343 | 0.099 |
| COX3 | Spawning salinity | -0.066 | 0.596 | -0.092 | 0.647 | -0.099 | 0.500 | -0.117 | 0.754 |
| Cytb | Spawning salinity | -0.128 | 0.755 | -0.157 | 0.795 | 0.190 | 0.205 | 0.236 | 0.152 |
| ND1 | Spawning salinity | -0.015 | 0.561 | -0.060 | 0.644 | NA | NA | NA | NA |
| ND2 | Spawning salinity | -0.153 | 0.771 | -0.162 | 0.785 | -0.320 | 0.954 | -0.316 | 0.946 |
| ND3 | Spawning salinity | -0.169 | 0.761 | -0.309 | 0.927 | 0.147 | 0.290 | 0.132 | 0.296 |
| ND4 | Spawning salinity | -0.092 | 0.661 | -0.125 | 0.738 | 0.053 | 0.385 | 0.047 | 0.393 |
| ND4L | Spawning salinity | 0.077 | 0.323 | 0.116 | 0.272 | -0.115 | 0.757 | -0.139 | 0.811 |
| ND5 | Spawning salinity | -0.099 | 0.763 | -0.124 | 0.780 | -0.313 | 0.933 | -0.330 | 0.949 |
| ND6 | Spawning salinity | -0.268 | 0.925 | -0.229 | 0.902 | -0.185 | 0.754 | -0.300 | 0.973 |
| CR | Spawning salinity | 0.007 | 0.451 | -0.015 | 0.511 | NA | NA | NA | NA |
| Genome | Spawning temp | -0.029 | 0.601 | -0.030 | 0.611 | NA | NA | NA | NA |
| Genes | Spawning temp | 0.000 | 0.514 | 0.000 | 0.523 | -0.149 | 0.819 | -0.146 | 0.811 |
| ATP6 | Spawning temp | -0.009 | 0.558 | -0.016 | 0.570 | 0.207 | 0.218 | 0.205 | 0.210 |
| ATP8 | Spawning temp | 0.230 | 0.154 | 0.230 | 0.153 | 0.457 | 0.073 | 0.465 | 0.073 |
| COX1 | Spawning temp | -0.209 | 0.877 | -0.209 | 0.876 | -0.127 | 0.740 | -0.132 | 0.755 |
| COX2 | Spawning temp | -0.019 | 0.462 | -0.017 | 0.462 | -0.136 | 0.858 | -0.141 | 0.845 |
| COX3 | Spawning temp | 0.004 | 0.455 | 0.004 | 0.450 | -0.039 | 0.564 | -0.039 | 0.557 |
| Cytb | Spawning temp | 0.113 | 0.273 | 0.113 | 0.273 | 0.345 | 0.038 | 0.345 | **0.035** |
| ND1 | Spawning temp | -0.012 | 0.537 | -0.014 | 0.553 | NA | NA | NA | NA |
| ND2 | Spawning temp | -0.129 | 0.747 | -0.128 | 0.746 | -0.178 | 0.835 | -0.175 | 0.819 |
| ND3 | Spawning temp | 0.390 | **0.021** | 0.388 | **0.022** | 0.073 | 0.262 | 0.071 | 0.274 |
| ND4 | Spawning temp | -0.045 | 0.579 | -0.046 | 0.578 | -0.207 | 0.864 | -0.208 | 0.851 |
| ND4L | Spawning temp | 0.313 | **0.030** | 0.314 | **0.029** | 0.173 | 0.128 | 0.173 | 0.126 |
| ND5 | Spawning temp | 0.035 | 0.433 | 0.035 | 0.434 | -0.059 | 0.565 | -0.056 | 0.553 |
| ND6 | Spawning temp | -0.050 | 0.623 | -0.046 | 0.612 | -0.145 | 0.800 | -0.148 | 0.795 |
| CR | Spawning temp | -0.159 | 0.856 | -0.160 | 0.852 | NA | NA | NA | NA |
| Genome | Latitude | 0.147 | 0.133 | 0.169 | 0.150 | NA | NA | NA | NA |
| Genes | Latitude | 0.145 | 0.129 | 0.177 | 0.136 | -0.094 | 0.747 | -0.027 | 0.566 |
| ATP6 | Latitude | 0.139 | 0.170 | 0.025 | 0.439 | 0.160 | 0.145 | 0.084 | 0.352 |
| ATP8 | Latitude | 0.185 | 0.122 | 0.033 | 0.432 | 0.138 | 0.170 | -0.018 | 0.570 |
| COX1 | Latitude | -0.035 | 0.594 | -0.023 | 0.559 | 0.017 | 0.447 | -0.058 | 0.641 |
| COX2 | Latitude | -0.051 | 0.622 | -0.025 | 0.553 | -0.167 | 0.782 | -0.282 | 0.918 |
| COX3 | Latitude | 0.128 | 0.202 | 0.149 | 0.214 | 0.159 | 0.182 | 0.201 | 0.186 |
| Cytb | Latitude | 0.101 | 0.238 | 0.129 | 0.221 | -0.076 | 0.686 | -0.099 | 0.700 |
| ND1 | Latitude | 0.151 | 0.140 | 0.149 | 0.190 | NA | NA | NA | NA |
| ND2 | Latitude | 0.006 | 0.474 | 0.036 | 0.421 | 0.032 | 0.408 | 0.122 | 0.268 |
| ND3 | Latitude | 0.368 | **0.007** | 0.373 | **0.013** | -0.119 | 0.749 | -0.192 | 0.837 |
| ND4 | Latitude | 0.097 | 0.250 | 0.113 | 0.260 | -0.079 | 0.685 | -0.114 | 0.724 |
| ND4L | Latitude | 0.063 | 0.306 | 0.089 | 0.292 | 0.100 | 0.370 | 0.129 | 0.314 |
| ND5 | Latitude | 0.199 | 0.065 | 0.244 | 0.074 | -0.009 | 0.520 | 0.052 | 0.400 |
| ND6 | Latitude | 0.088 | 0.247 | 0.209 | 0.080 | -0.003 | 0.492 | -0.055 | 0.625 |
